# Causes of variation among rice models in yield response to CO_2_ examined with Free-Air CO_2_ Enrichment and growth chamber experiments

**DOI:** 10.1038/s41598-017-13582-y

**Published:** 2017-11-01

**Authors:** Toshihiro Hasegawa, Tao Li, Xinyou Yin, Yan Zhu, Kenneth Boote, Jeffrey Baker, Simone Bregaglio, Samuel Buis, Roberto Confalonieri, Job Fugice, Tamon Fumoto, Donald Gaydon, Soora Naresh Kumar, Tanguy Lafarge, Manuel Marcaida III, Yuji Masutomi, Hiroshi Nakagawa, Philippe Oriol, Françoise Ruget, Upendra Singh, Liang Tang, Fulu Tao, Hitomi Wakatsuki, Daniel Wallach, Yulong Wang, Lloyd Ted Wilson, Lianxin Yang, Yubin Yang, Hiroe Yoshida, Zhao Zhang, Jianguo Zhu

**Affiliations:** 1 Tohoku Agricultural Research Center, National Agriculture and Food Research Organization, Morioka, Iwate, 020-0198 Japan; 2 International Rice Research Institute, DAPO Box, 7777 Metro Manila, Philippines; 3 Centre for Crop Systems Analysis, Wageningen University & Research, Wageningen, PO Box 430 The Netherlands; 4 National Engineering and Technology Center for Information Agriculture, Nanjing Agricultural University, Nanjing, Jiangsu 210095 China; 5 Key Laboratory for Crop System Analysis and Decision Making, Ministry of Agriculture, Nanjing Agricultural University, Nanjing, Jiangsu 210095 China; 6 Jiangsu Key Laboratory for Information Agriculture, Nanjing Agricultural University, Nanjing, Jiangsu 210095 China; 7 Jiangsu Collaborative Innovation Center for Modern Crop Production, Nanjing Agricultural University, Nanjing, Jiangsu 210095 China; 8 University of Florida, Gainesville, Florida, 32611-0500 USA; 9 United States Department of Agriculture, Agricultural Research Service, Big Spring, Texas 79720 USA; 10 CREA, Research Center for Agriculture and Environment, Bologna, 40128 Italy; 11 INRA, UMR1114 EMMAH, F-84914 Avignon, France; 12 University of Milan, Cassandra lab, Milan, 20133 Italy; 13 International Fertilizer Development Center, Muscle Shoals, Alabama 35662 USA; 14 Institute for Agro-Environmental Sciences, National Agriculture and Food Research Organization, Tsukuba, Ibaraki, 305-8604 Japan; 15 CSIRO Agriculture and Food, St Lucia, QLD 4067 Australia; 16 Indian Agricultural Research Institute, New Delhi, 110012 India; 17 Cirad, UMR AGAP, F-34398 Montpellier, France; 18 AGAP, Univ Montpellier, CIRAD, INRA, INRIA, Montpellier SupAgro, Montpellier, France; 19 Ibaraki University, College of Agriculture, Inashiki, Ibaraki, 300-0393 Japan; 20 Chinese Academy of Sciences, Institute of Geographical Sciences and Natural Resources Research, Beijing, 100101 P.R. China; 21 Natural Resources Institute Finland (Luke), FI-00790 Helsinki, Finland; 22 INRA, UMR AGIR, Castanet Tolosan, France; 23 Yangzhou University, Hanjiang, Yangzhou, Jiangsu 225009 China; 24 Texas A&M AgriLife Research Center, Beaumont, Texas 77701 USA; 25 State Key Laboratory of Earth Surface Processes and Resource Ecology, Beijing Normal University, Beijing, 100875 P.R. China; 26 State Key Laboratory of Soil and Sustainable Agriculture, Institute of Soil Sciences, Chinese Academy of Sciences, Nanjing, 210008 China

## Abstract

The CO_2_ fertilization effect is a major source of uncertainty in crop models for future yield forecasts, but coordinated efforts to determine the mechanisms of this uncertainty have been lacking. Here, we studied causes of uncertainty among 16 crop models in predicting rice yield in response to elevated [CO_2_] (E-[CO_2_]) by comparison to free-air CO_2_ enrichment (FACE) and chamber experiments. The model ensemble reproduced the experimental results well. However, yield prediction in response to E-[CO_2_] varied significantly among the rice models. The variation was not random: models that overestimated at one experiment simulated greater yield enhancements at the others. The variation was not associated with model structure or magnitude of photosynthetic response to E-[CO_2_] but was significantly associated with the predictions of leaf area. This suggests that modelled secondary effects of E-[CO_2_] on morphological development, primarily leaf area, are the sources of model uncertainty. Rice morphological development is conservative to carbon acquisition. Uncertainty will be reduced by incorporating this conservative nature of the morphological response to E-[CO_2_] into the models. Nitrogen levels, particularly under limited situations, make the prediction more uncertain. Improving models to account for [CO_2_] × N interactions is necessary to better evaluate management practices under climate change.

## Introduction

Climate change is a daunting challenge to world agriculture in meeting the demand for food and energy from a growing population^1^. The production of rice, a staple food crop in Asia, is strongly affected by climate change^2^. Atmospheric CO_2_ concentration ([CO_2_]) is an important driver for climate change. The most recent projections adopted by the Intergovernmental Panel on Climate Change show that [CO_2_] may reach as high as about 540 μmol mol^−1^ by 2050 and 940 μmol mol^−1^ by 2100^3^ and will have significant impacts on global climate systems.

On the other hand, rising [CO_2_] will have some positive effects on the productivity of C_3_ crops like rice, by increasing photosynthesis, biomass and grain yield. This CO_2_ fertilization will alleviate some of the negative effects of increasing temperatures^4^, but prior studies suggest that the magnitude of the CO_2_ fertilization effect is highly uncertain^5,6^.

Crop models are pivotal tools for assessing the impact of climate change variables^7^, but models differ in their descriptions of processes and forcing variables^8^. Even for the major processes such as photosynthesis and biomass production that are directly affected by elevated [CO_2_] (E-[CO_2_]), some models use the coarse-grained concept of radiation use efficiency (RUE) whereas other models use a light response curve (LRC) of instantaneous leaf photosynthesis scaled to hourly or daily canopy photosynthesis and crop respiration. Some crop models simulate instantaneous leaf photosynthesis by use of the biochemical model of Farquhar, von Caemmerer & Berry (FvCB)^9^. Such diversity in model algorithms, combined with inconsistency in model parameterization procedures, can create a large range of uncertainties in model projections^10–13^.

Another direct effect of E-[CO_2_] is on stomatal conductance and transpiration, which could affect crop production under water limited environments^14^. However, because rice is generally grown in fields where 5–10 cm of floodwater is maintained, the effect of E-[CO_2_] on crop yield via stomatal conductance for irrigated rice is in general considered to be negligible. We therefore hypothesized that the difference in model types for the primary response of photosynthesis and biomass production to E-[CO_2_] creates uncertainties in yield prediction.

Coordinated crop model intercomparison has recently become feasible through the Agricultural Model Intercomparison and Improvement Project (AgMIP)^15^. Our initial attempt has shown that rice crop models differ considerably in yield and biomass responses to [CO_2_]^12^, but the sources of uncertainties were not fully analyzed at that time, particularly lacking comparison with experimental observations under E-[CO_2_]. There is an urgent need to identify the sources of uncertainty and to improve the methods of prediction of the effects of E-[CO_2_]^6^.

Two types of experimental facilities have been exploited to determine the effects of E-[CO_2_] on various plant traits; small-scale growth enclosures or chambers^16^ and large-scale field environments using FACE (Free-Air CO_2_ Enrichment)^17^. There are arguments, however, that the experimentally observed CO_2_ enhancement on yield and other plant traits differ between FACE and enclosed chambers^18,19^, suggesting that uncertainty also exists in the observations and that testing multiple models against multiple sources of experimental results is desirable.

The CO_2_ fertilization effects on rice yield are not constant and also depend on other factors such as temperature^20,21^ and nitrogen (N) availability^22,23^. Most rice models take account of these factors, but differences in how crop models simulate [CO_2_] × N interactions are yet to be quantified. In this study, we compiled three years of FACE data conducted at two sites with contrasting climate conditions: one in northern Japan with a cool temperate climate^22^ and the other in central China with a subtropical climate. Each site had three N levels to test the [CO_2_] × N interactions. We also compiled results from a series of Soil-Plant-Atmosphere-Research (SPAR) chamber experiments1^6,24^. The SPAR chamber experiments conducted in Gainesville, Florida, USA, included six levels of [CO_2_], so are suitable to test model responses to various levels of [CO_2_]. Another SPAR experiment with three levels of N was conducted to test the [CO_2_] × N interactions. With these datasets and collectively 16 rice models, uncertainty analyses using a multi-model ensemble and multi-experimental facilities are possible.

Our objectives are: (i) to determine the variation of yield prediction among existing commonly used rice models of different types, (ii) to identify the processes/mechanisms/factors that cause this uncertainty, (iii) to analyze to what extent the models differ in simulating [CO_2_] × N interactions, and (iv) to propose possible directions for model improvement.

## Results

### Modelled primary responses to changes in [CO_2_]

**Figure 1:**
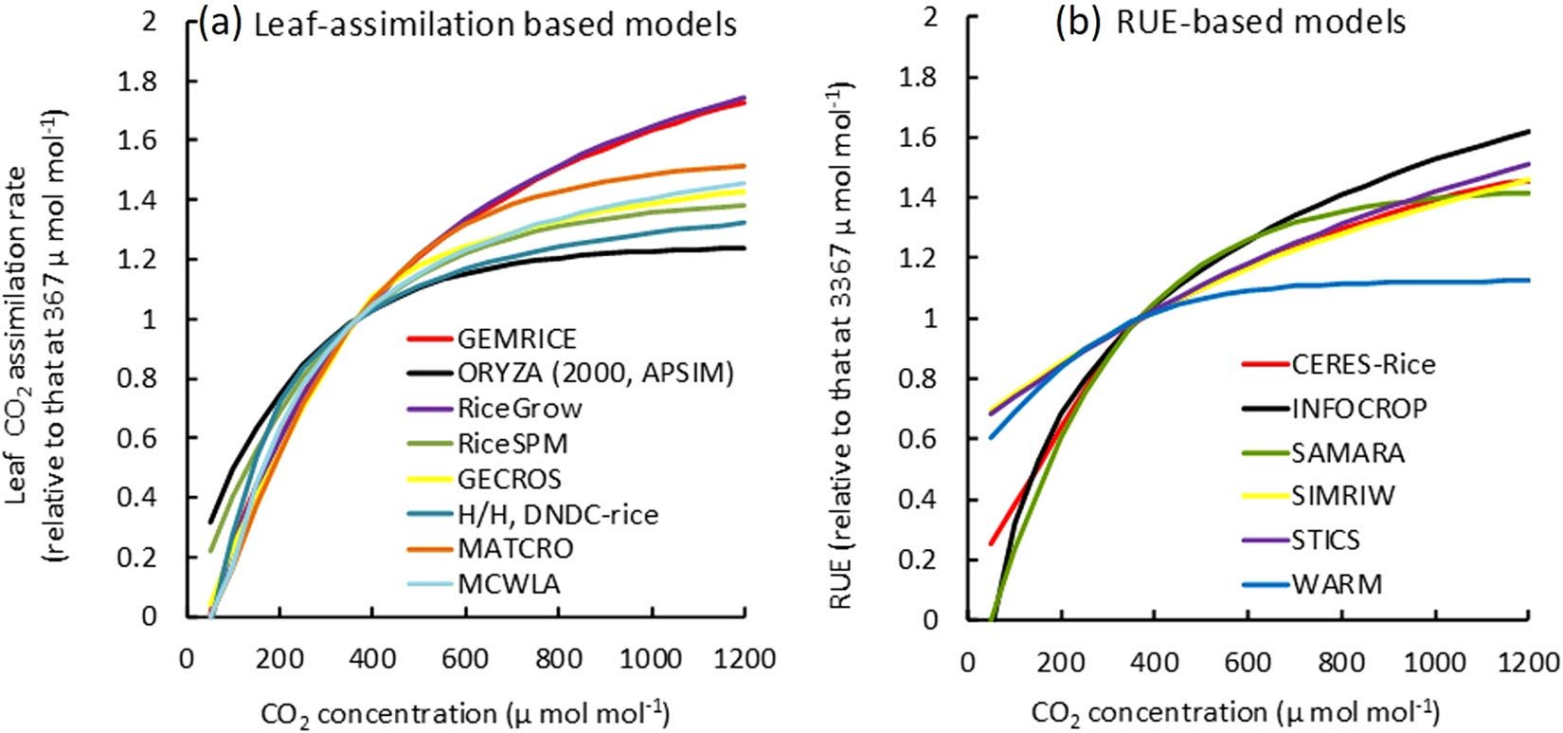
[CO_2_] response curves used for (**a**) leaf CO_2_ assimilation rate or (**b**) radiation use efficiency used in 16 rice models (Primary CO_2_ response). Values were scaled to that at 367 µmol mol^−1^ (average daytime ambient [CO_2_] in the FACE experiments). Each response was estimated under the following conditions: Photosynthetically active radiation, 2000 µmol m^2^-s^−1^; Relative humidity, 70%, Air temperature, 25 °C; Wind speed, 1 ms^−1^, Leaf N content, 2 gm^−2^; Leaf N concentration, 40 mg g^−1^; Specific leaf mass, 200 cm^2^ g^−1^.

All 16 models listed in Table 1 simulate leaf area index, canopy light interception, biomass production and grain yield^12,13^ and account for the direct effect of E-[CO_2_] on photosynthetic rates or canopy radiation use efficiency (RUE). All models have a non-linear asymptotic response of leaf photosynthetic rate or canopy radiation use efficiency (RUE) to changes in [CO_2_], but the type of algorithm used varies (Table 1, Methods) and the responses differ in magnitude (Fig. 1). At 567 µmol mol^−1^ (averaged [CO_2_] for all FACE treatments), increases in assimilation or RUE relative to that at 367 μmol mol^−1^ (average daytime ambient [CO_2_] in the FACE experiments) ranged from 14 to 30% for leaf photosynthesis and from 8 to 24% for canopy level RUE response (Fig. 1). Averaged responses were similar across different model types (16–20%), which agreed well with the summary of observations in the FACE and chamber experiments 20.

**Table 1:** Models used for the simulation exercise and their main characteristics.

Model	Exercise simulated		CO_2_ response for primary production			Other direct effects of [CO_2_]	Leaf area increase			Yield formation			Reference
	FACE	SPAR	Leaf-level		Canopy-level		Resource-driven		Temperature-driven	Direct CO_2_ effect on grain set or harvest index	Grain number	Partitioning coefficient	
			LRC	FvCB	RUE		Carbon	Nitrogen					
1. APSIM-ORYZA	●	●	●				●				●		47,48
2. CERES-RICE	●	●			●	Gs, Tr			●		●		49
3. DNDC-Rice	●	●		●		Gs, Tr		●				●	20,50,51
4. GECROS	●	●		●		Gs, Gm, Tr	●	●			●		52
5. GEMRICE	●	●	●			phenology, spikelet sterility			●	●	●		53–56
6. H/H	●	●		●		Gs		●				●	20,51,57
7. InfoCrop		●			●	Tr,	●	●			●		58
8. MATCRO^§^	●	●		●		Gs	●					●	59–61
9. MCWLA^§^	●	●		●		Gs, Tr			●	●		●	62
10. ORYZA2000	●	●	●				●				●		47
11. RiceGrow	●	●	●				●					●	63
12. RicePSM	●	●	●				●	●			●		64
13. SAMARA^§^		●			●	Tr			●		●		65
14. SIMRIW^§^	●	●			●	phenology, spikelet sterility			●	●		●	66,67
15. STICS	●				●	Gs, Tr			●		●	●	68,69
16. WARM^§^	●	●			●		●					●	70,71

Gs, stomatal conductance; Gm, mesophyll conductance; Tr, transpiration.

Leaf area increase;

Resource-driven, dependent: LAI increases with the resource such as C and N allocated to the leaves;

Temperature-driven, LAI increases without any effects of resource availability (only as a function of developmental stages or temperatures).

Yield formation;

Grain number, yield is calculated by grain number × individual grain weight;

Partitioning coefficient, yield is calculated by biomass × harvest index.;

Direct CO_2_ effect on grain set or harvest index, models that account for a direct effect of E-[CO_2_] on grain set or harvest index.

^§^The models that do not include the quantification of the effects of different nitrogen on crop growth and yield.

### Model simulations under ambient [CO_2_]

**Figure 2:**
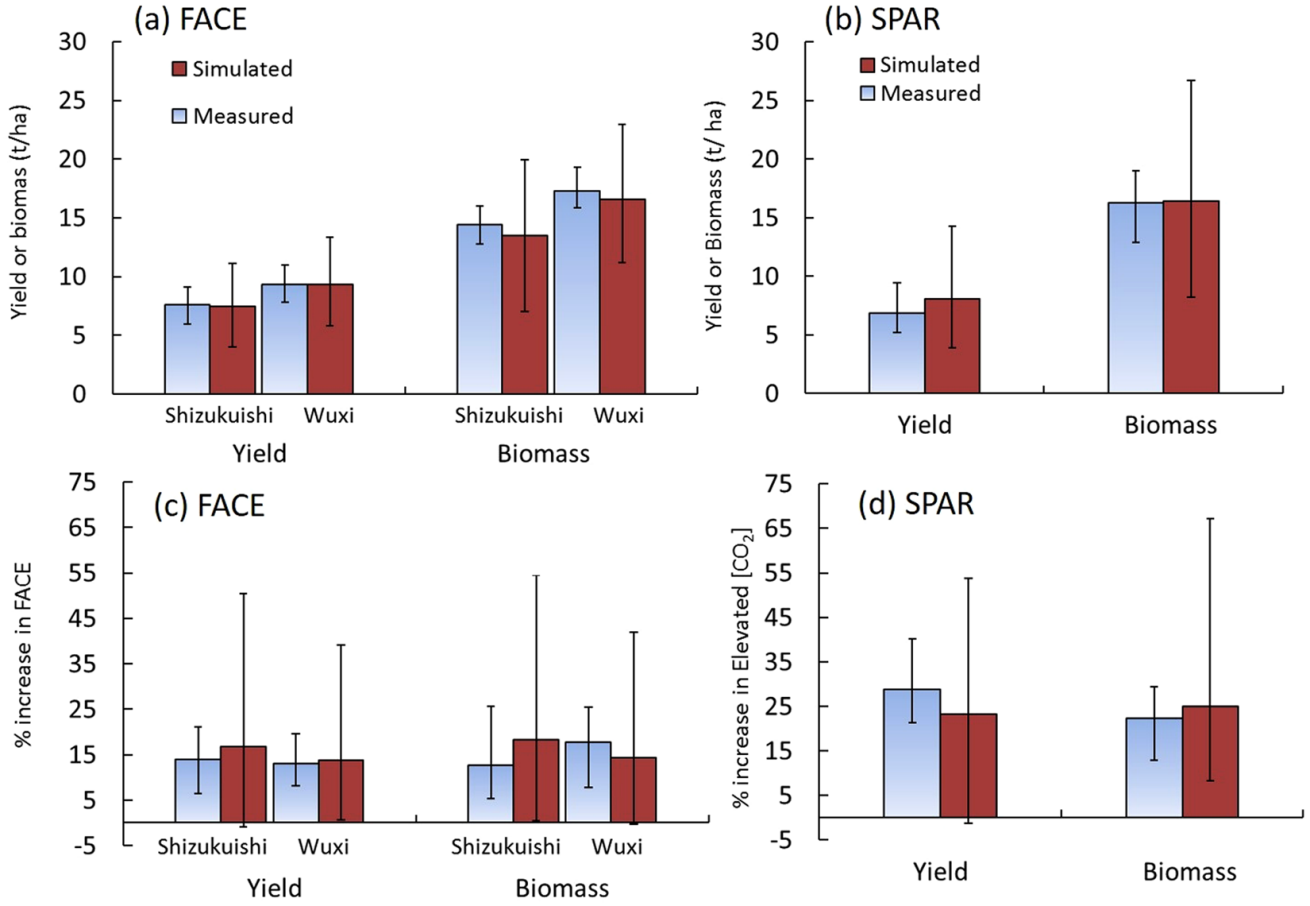
Simulated and observed yield and biomass. Grain yield and biomass under ambient [CO_2_] in (**a**) FACE and (**b**) SPAR experiments; % increase in yield and biomass in response to elevated [CO_2_] in (**c**) FACE and (**d**) SPAR experiments. Each bar represents average across different N treatments and years at two FACE sites left (Shizukuishi and Wuxi) and in the SPAR chamber experiments (Exp. 1, 2 and 3). Error bars represent the maximum and minimum values of each measurement or simulation. Data sets used for calibration are not included. For % increase in elevated [CO_2_] in the SPAR chambers (**d**), mean of values under 500 and 660 µmol mol^−1^ relative to ambient are presented. Comparison by treatment in the FACE experiments are detailed in Figures S2 and S3.

Models were first run under ambient [CO_2_] conditions and different N levels at the Shizukuishi (Japan) and Wuxi (China) FACE sites (Methods, Table S1). Observed yields of rice under ambient [CO_2_] were generally greater at Wuxi than at Shizukuishi because of the higher N fertilization rates, longer duration, and higher yield potential of the cultivar used at Wuxi (Methods). Simulated yields of all models under ambient [CO_2_] averaged 7.5 t ha^−1^ and 9.3 t ha^−1^ at Shizukuishi and Wuxi, respectively, in good agreement with the observed yields of 7.9 t ha^−1^ and 9.4 t ha^−1^ averaged over N treatments and years at each site (Fig. 2a). A pairwise T-test comparing simulations with observations for all data, excluding those used for model calibration, showed modestly significant differences in some instances, but the differences are less than the experimental standard errors (Detailed analyses are given in Tables S2 and S3). Model ensemble means agreed well with the observed means and variation in yield and biomass under different N and years (Figure S2). However, simulated yields of individual models for each N treatment ranged from 4.0 t ha^−1^ to 11.1 t ha^−1^ at Shizukuishi and from 5.8 t ha^−1^ to 13.3 t ha^−1^ at Wuxi (Fig. 2a). At both sites, yield and biomass varied substantially among years and N treatments but the model-to-model variation was the most important cause of variability, accounting for up to two-thirds of total variability (Table S5, Supplementary text for uncertainty analysis). The variance of the simulated yield and biomass among the models was 13–62 times greater than the residual variance of the experiments (Table S4).

Models were also run for reference [CO_2_] (330 µmol mol^−1^) for the SPAR chamber experiments conducted in Florida, USA (Methods). Simulated yield and biomass averaged over the three experiments (standard N only) differed from observed values by only 1.3 t/ha and 0.2 t/ha, respectively (Fig. 2b), and the differences were less than the standard error for the measurement mean (Table S5). The variance in simulated yield and biomass was mostly attributable to the variability between models (71% for grain yield and 57% for biomass, Table S5), and was 7–8 times greater than the variance between experiments (Tables S5, S6).

### Model simulations for the effects of elevated [CO_2_]

#### Model simulations for the yield enhancement due to E-[CO_2_]

Simulations were conducted under E-[CO_2_] conditions at the two FACE sites. The mean [CO_2_] in the FACE plots was 567 µmol mol^−1^ (Methods). The observed increase due to E-[CO_2_] on grain yield averaged 12.8% and was similar between the two sites: 12.4% at Shizukuishi and 13.3% at Wuxi (Fig. 2c). Simulated increase of grain yield due to E-[CO_2_] averaged for all models was slightly greater than the measurements, by about 3 and 1 percent point at Shizukuishi and Wuxi, respectively. These differences are less than the observed residual standard error (see Figure S3 and Table S2). However, simulated yield increase due to E-[CO_2_] varied considerably among models, treatments and years, ranging from -1 to 50% (Figs 2c, S4). Simulated increases in biomass in response to E-[CO_2_] also varied greatly depending on model and treatment. The ranges of simulated and observed increases were greater at Shizukuishi than at Wuxi (Figs 2c and S4).

**Figure 3:**
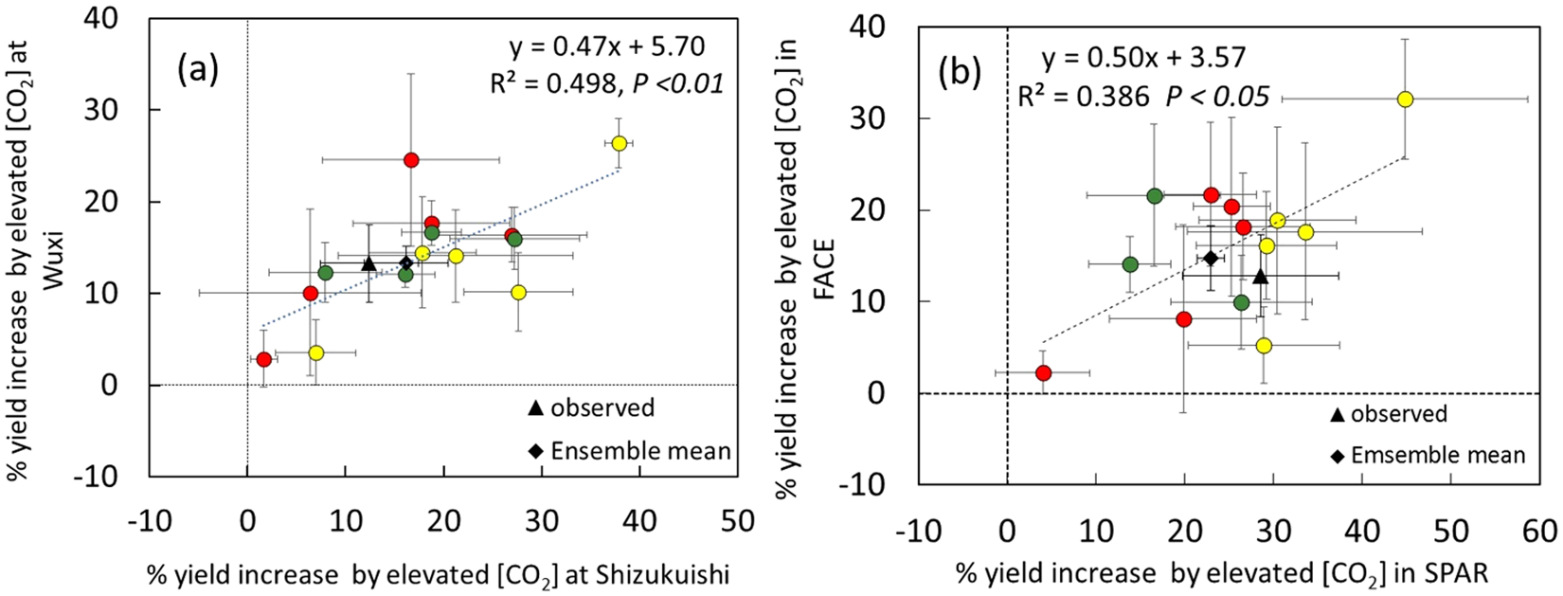
Comparison between yield response to elevated [CO_2_] of 14 individual rice models (**a**) between two FACE sites and (**b**) between FACE and SPAR chamber experiments. Each point is an average over different N treatments and years for FACE. Red, LRC (light response curve-type photosynthesis model); Yellow, FvCB (Farquhar, von Caemmerer & Berry photosynthesis model); Green, RUE (Radiation use efficiency). Means of the observed and simulated are also shown. FACE = mean value for Shizukuishi and Wuxi. SPAR = mean value for 500 and 660 µmol mol^−1^ (ex 1, 2 & 3). SD is the measure of variation within each model due to different conditions (N, years or site).

Between-model variation accounted for about 60-70% of the variation in simulated yield increase (Table S3). Simulated yield increases for individual models, averaged over treatments and years at each site, varied from 2 to 38% at Shizukuishi (Fig. 3a, X-axis) and from 3 to 26% at Wuxi (Fig. 3a, Y-axis). Simulated yield increase due to E-[CO_2_] at Shizukuishi was significantly correlated with that at Wuxi (*P* < 0.01, Fig. 3a) so that models that overestimated at one site also simulated greater yields at the other site, suggesting that the magnitude of simulated CO_2_ fertilization effects by each model was consistent across the sites.

No clear difference was observed between model types, but substantial variation exists within each model type (Figs 3a and S4), suggesting that the model structure for primary CO_2_ response is not the major source of variation.

Simulated yield and biomass by each model greatly differed among treatments (Figures S3, S4). However, the between-model variation accounted for about 30 to 70% of the total variation in simulated yields, suggesting that within- model variation is of major importance (Table S4).

Simulated yield and biomass increases in response to E-[CO_2_] in the SPAR experiments (mean of values at 500 and 660 µmol mol^−1^, comparable to the change of [CO_2_] in the FACE studies), were also similar to the measured increases (Fig. 2d). The observed and simulated yield increases were generally greater in the SPAR chamber than in the FACE studies (Fig. 2c,d), although the simulations slightly overestimated the FACE response and underestimated the chamber response.

A significantly positive correlation exists in the modelled yield response between FACE and chamber studies (Fig. 3b, *P* < 0.05). This result supports the consistency of model performances under different experimental setups.

#### Model simulations for the yield response to various [CO_2_]

**Figure 4:**
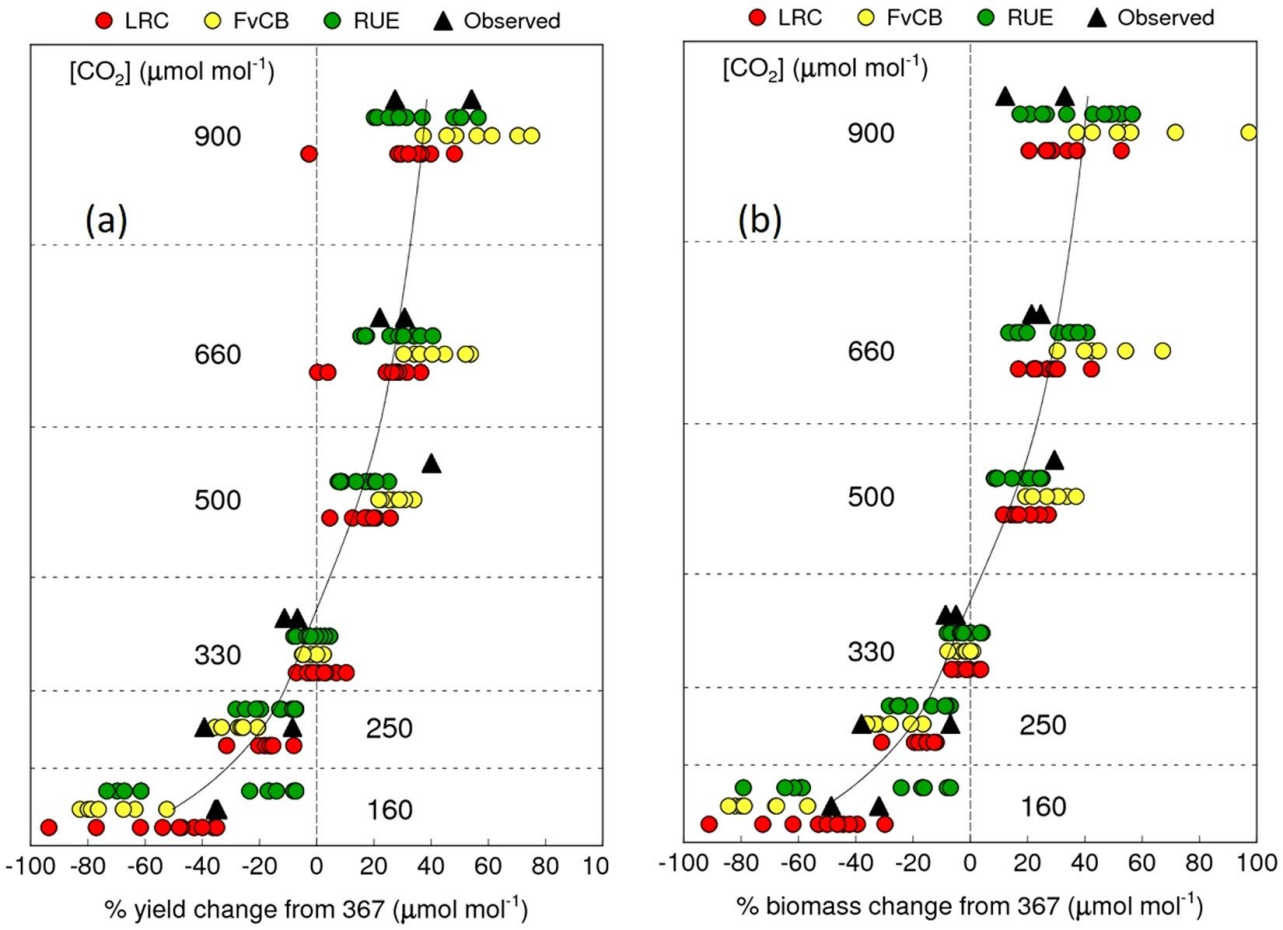
Simulated and observed response of (**a**) grain yield and (**b**) aboveground biomass to six [CO_2_] levels. LRC, light response curve-type photosynthesis model; FvCB, Farquhar, von Caemmerer & Berry photosynthesis model; RUE, radiation use efficiency. Numbers in the figures represent the growth [CO_2_] conditions. % changes are relative to the value at 367 µmol mol^−1^. Solid curve represents the model ensemble mean.

The SPAR chamber experiments tested six levels of [CO_2_], ranging from 160 to 900 µmol mol^−1^ for two growing seasons. The observed yields showed a typical asymptotic response to increasing [CO_2_]: grain yield increased more with increasing [CO_2_] within the sub-ambient [CO_2_] range than the supra-ambient range, and above 500 µmol mol^−1^ yield plateaued to a similar level of around 10 to 40% increase relative to yield at [CO_2_] at 367 µmol mol^−1^ (Fig. 4a). Model ensemble means agreed well with the response observed at different [CO_2_] but with a large variation between different models. There was no clear difference between model types, although FvCB-type models tended to be more sensitive (responsive) to [CO_2_]. Biomass followed a response pattern similar to grain yield with a similar variation among models (Fig. 4b).

#### Model simulations for the yield response to various [CO_2_] under different N levels

**Figure 5:**
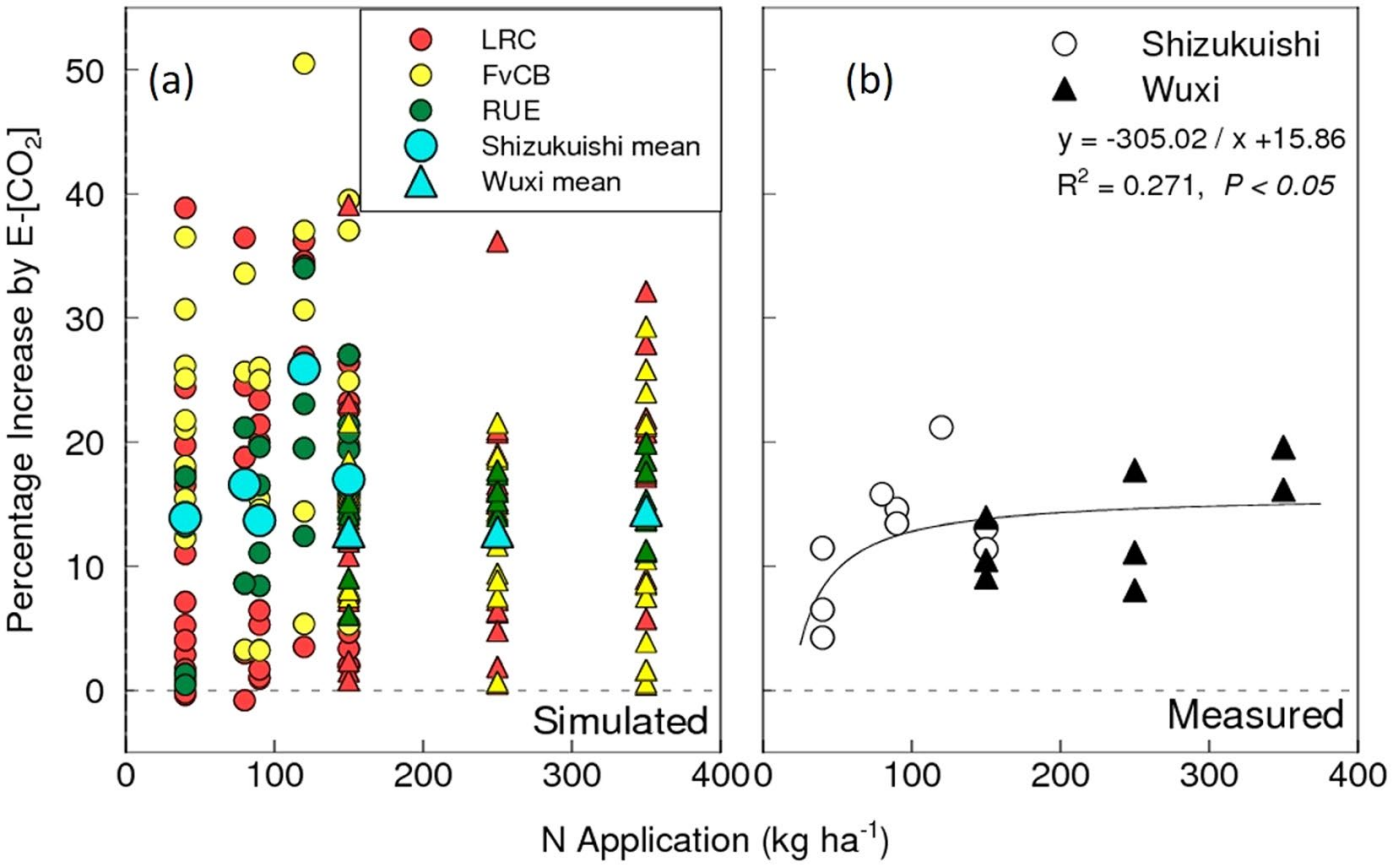
Simulated (**a**) and observed (**b**) yield enhancements due to elevated [CO_2_] under different N levels obtained at Shizukuishi FACE site in Japan and Wuxi site in China. LRC, light response curve-type photosynthesis model; FvCB, Farquhar, von Caemmerer & Berry photosynthesis model; RUE, radiation use efficiency. Note that five out 14 models do not take N limitation into account (Table 1) and simulated only under the highest N conditions at each site.

Both FACE sites had three levels of N applications. However, because the N doses were based on the conventional practices at each site, they differed between the two sites (Methods). Nevertheless, the effect of [CO_2_] fertilization on yield was similar at both sites, with the observed yield enhancement being smaller when N supply was limited (Fig. 5), as reported by Kim *et al*.^22^ for Shizukuishi and Yang *et al*.^23^ for Wuxi. However, simulated yield enhancement did not show a sufficient degree of dependence on N levels (Fig. 5). At all N levels, variation in yield response among models was large but variation tended to be greater at lower N levels. There was no clear difference between the model types in magnitude or N dependence of the yield enhancements.

One of the SPAR experiments (Experiment 3) compared factorial combinations of two [CO_2_] and three N levels. Enhancements due to E-[CO_2_] were not different among N levels (Figure S5) for either simulated or observed yields. Model-to-model variation in yield response to E-[CO_2_] was generally smaller at the highest N levels and increased as N decreased. No clear difference was observed between model types.

### Factors influencing the variation in simulated [CO_2_] response

**Figure 6:**
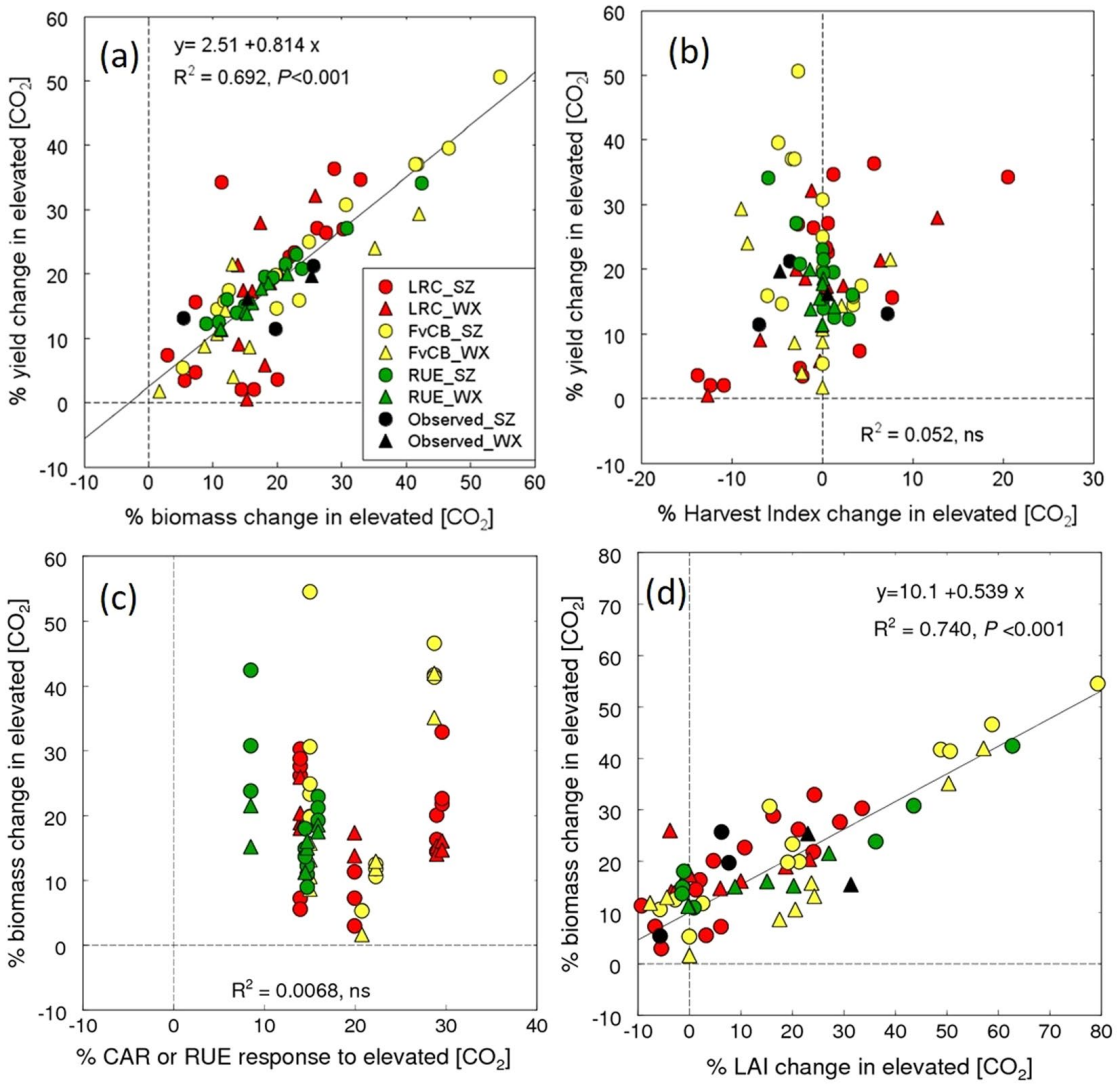
Factors affecting the simulated increase in grain yield due to E-[CO_2_] in the high N treatment in the FACE experiments at two sites. (**a**) grain yield increase versus biomass increase, (**b**) grain yield increase versus harvest index increase, (**c**) biomass increase versus primary [CO_2_] increase (leaf CO_2_ assimilation rate, CAR, or radiation use efficiency, RUE), and (**d**) biomass increase versus maximum LAI increase. The data from all N treatments and the chamber experiments are shown in Figures S6–10. LRC, light response curve-type photosynthesis model; FvCB, Farquhar, von Caemmerer & Berry photosynthesis model; RUE, radiation use efficiency. SZ = Shizukuishi, WX = Wuxi.

Simulated yield response to E-[CO_2_] was positively correlated with the simulated biomass response for both FACE (Figs 6a, S6a, *P* < 0.001) and SPAR chamber studies (Figure S7, P < 0.001). About 69% of the variation in the simulated yield increase in the FACE studies and 67–97% in the SPAR chamber experiments was attributed to the simulated response in biomass. On the other hand, variation among models in simulated harvest index was not or much less correlated with yield response than that in biomass response (Figs 6b, S6b, S8).

To identify factors affecting biomass increase, we examined the relationship between primary [CO_2_] response of the model (Fig. 1) and simulated biomass increase (Figs 6c, S6c and S9), because this is the primary direct mechanism by which modelled biomass changes in response to different [CO_2_]. However, variation in simulated primary response to different [CO_2_] was not correlated with biomass response, except in sub-ambient [CO_2_] treatments. A weak but significantly positive correlation was observed between primary response and biomass response to 160 and 250 µmol mol^−1^ (Figure S9d and e).

Another determinant of biomass production is canopy light interception, which is largely determined by leaf area index (LAI). The variation in the biomass response to [CO_2_] was closely and positively correlated with that of the maximum LAI response in both FACE and SPAR experiments and at all [CO_2_] levels (Figs 6d, S6d and S10). Models differ in their LAI submodules and can be classified into four types (Table 1). Some models use a resource-driven approach for LAI growth, the classic approach being carbon (or biomass)-dependent modules. In this approach, carbon allocated to the leaves is converted to leaf area based on fixed area-to-weight ratios for leaf (C-dependent type). Other models use N as a key driver for leaf growth, in which leaf area increases in proportion to N uptake, or N allocated to the leaves (N-dependent type). In some models, leaf area growth is a function of temperature, where, for instance, leaf area increases in proportion to growing degree days, regardless of C or N availability (Resource-independent, temperature-driven type). Some models use the carbon-driven approach combined with other limitations.

**Figure 7:**
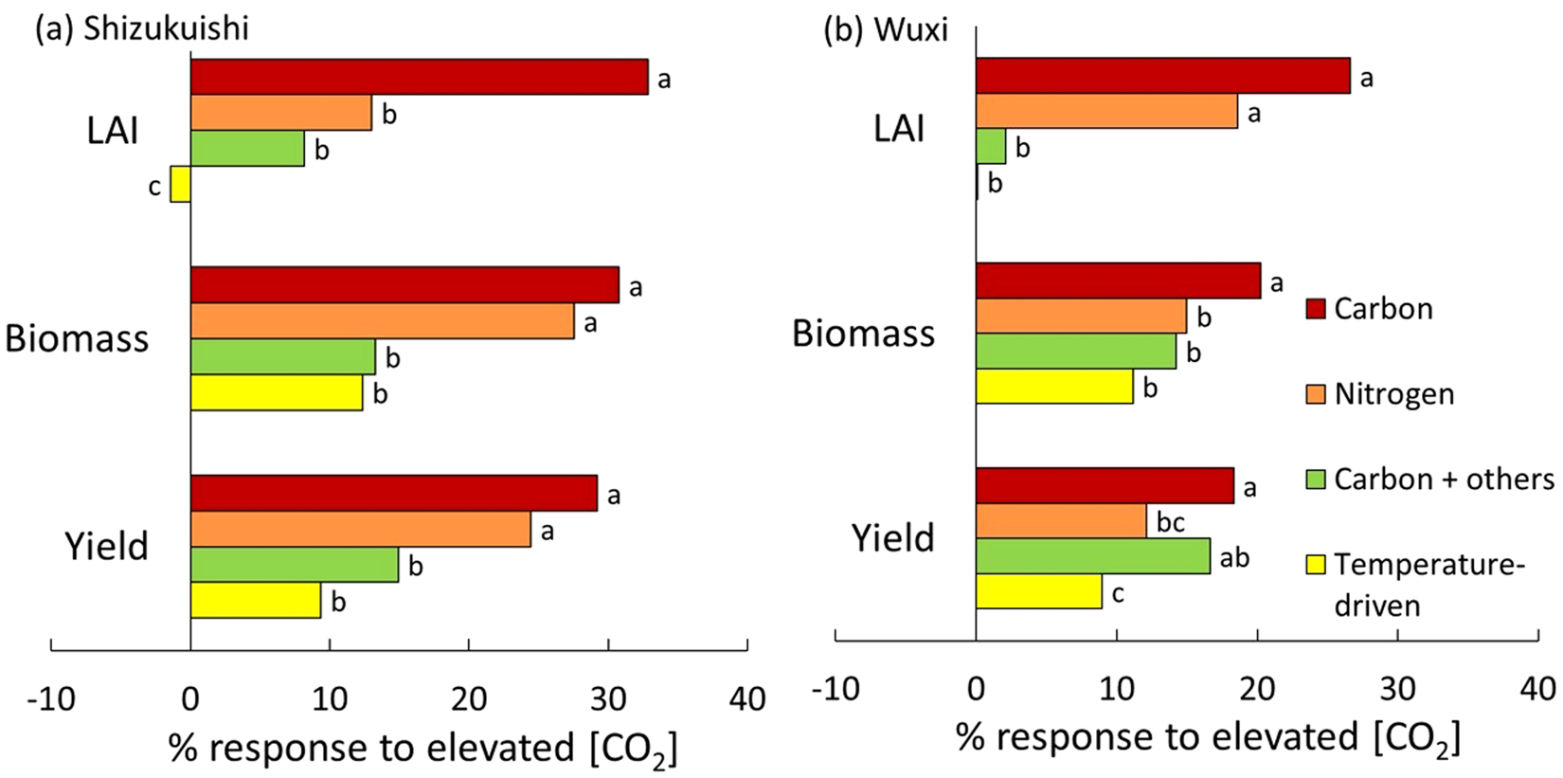
Simulated response of maximum LAI, final biomass and grain yield at Shizukuishi (**a**) and Wuxi (**b**) for models with different approaches to calculating LAI. Data are expressed as a ratio to response at 367 µmol mol^−1^. At each site, all variables are significantly different between model types (*P* < 0.05 for biomass at Wuxi and *P* < 0.001 for all other variables). The main effect of N or interactions between N and model type were not significant for any variables. Letters with different letters beside the bar indicate that the between-group is different at *P* = 0.05, by the Tukey b method (Wholly Significant Difference).

There were significant differences in the LAI response to E-[CO_2_] among the model types. Carbon-dependent models showed as much as a 30% increase in maximum LAI in response to E-[CO_2_], whereas no increase was recorded with the temperature-driven (resource-independent) type (Fig. 7). The other two types had intermediate responses. The large variation in maximum LAI response resulted in significant differences in responses in biomass and yield.

## Discussion

Crop response to increasing [CO_2_] has been identified as a major source of uncertainty in predicting crop production 6. In this study, we confirmed substantial variation in yield prediction in response to E-[CO_2_] among rice crop models, which was much greater than the experimental variation. Here we used data from FACE and SPAR chamber experiments to identify the sources of uncertainty. Experiments with small pots could limit the response to E-[CO_2_]^25^, but the SPAR chamber experiments used large containers containing soil of 50 cm depth, so the effect of the limited rooting zone was assumed to be minimized.

Despite the large differences between models, the mean computed over all model simulations was close to the observed yield and yield response to E-[CO_2_], as previously reported^10–12,26^. The observed yield increase in response to E-[CO_2_] was greater in the chamber studies than in the FACE study, in agreement with the meta-analysis by Long *et al*.^5^. However, the mean over all models reproduced similar differences in yield response between FACE and chamber studies, although the mean slightly overestimated the FACE results and underestimated the chamber results. The reason why simulations followed the yield responses of both experimental setups could be partly due to growth conditions (temperature and N fertilization) in the SPAR chambers, with ample N under warmer air being beneficial for the CO_2_ fertilization effects, compared to the two FACE studies. Such a difference could have caused the greater yield response to E-[CO_2_] in the SPAR chambers and to some extent in the simulations. There are arguments as to whether results from FACE and enclosure studies are intrinsically different^5,27–29^, but the present study suggests that the differences between the two technologies are due to differences in environmental and management factors and that model ensembles can reproduce these differences.

Differences in predictions among models were relatively consistent between the two sites (Fig. 3a) and between FACE and chamber studies (Fig. 3b), unlike a case study on wheat^26^. Models that tend to overestimate the increase in one environment are likely to overestimate in the other environments, which suggests that model uncertainties are not random, and that opportunities exist for reduction in model uncertainty through identifying the mechanisms underlying the consistent biases.

Yield is a result of biomass production and allocation to harvestable organs (harvest index). The present study showed that much of the variation in yield resulted from variation in total biomass. The primary response to [CO_2_] is the direct mechanism by which biomass is influenced in different [CO_2_] conditions, and the models differ in complexity or mechanisticity for the primary response. However, the model type (LRC, FvCB or RUE) did not account for differences in simulated biomass response to E-[CO_2_] (Figs 6c and S9), which was not surprising since the magnitude of the primary response to E-[CO_2_] did not differ among model types (17–21%, Figure S1).

Model primary response to E-[CO_2_] (+200 µmol mol^−1^) ranged from 8 to 30% among models (Fig. 1), but was not related to simulated biomass response to E-[CO_2_]. On the other hand, simulated LAI in response to E-[CO_2_] accounted for much of the model-to-model variation in the biomass response to E-[CO_2_] (Figs 6d,S6d and S10 despite the fact that none of the models included direct mechanisms by which E-[CO_2_] influences leaf area expansion. Nevertheless, increase in LAI in response to E-[CO_2_] varied substantially among the models. This is in contrast to a recent study testing six wheat models against Australian FACE data^30^, which found similar LAI increases among models of 7–24% due to E-[CO_2_]. The 16 rice models tested in this study differed in LAI formulations: for instance, some models simulate LAI increase assuming leaf growth is limited by carbon allocated to the leaves, whereas some use a function of temperature only (Table 1). Because carbon gain is enhanced by E-[CO_2_], LAI in the C-driven model is increased by E-[CO_2_]. As a result, light interception increases, which enhances biomass production. This positive feedback results in a biomass increase that is much greater than the primary [CO_2_] response. On the other hand, the advantage of greater light interception does not occur with resource-independent type models, so the biomass response is largely determined by the primary response to E-[CO_2_]. In an earlier study with wheat, Ewert^31^ pointed out the importance of accurate estimation of LAI for simulating the effect of [CO_2_] on canopy carbon gain. The present study shows that much more of the between-model variability in biomass in rice can be attributed to variability in simulated leaf area than to variability in primary response to E-[CO_2_].

Variation in simulated leaf area among models can also be a source of uncertainty in estimating gas and energy exchange between land surface and atmosphere, which is one of the most important feedbacks to the climate system. Because agricultural land represents a large segment of terrestrial ecosystems, accurate estimation of gas and heat exchange in croplands is important. Crop canopy cover or LAI is one of the most important state variables for the gas and heat exchange process, but its response to climate change is a major source of uncertainty. Improving models to reduce the uncertainty in leaf area estimation is thus also important for the modelling terrestrial energy and water cycles.

Experimental observations in the FACE experiments showed that the response of maximum LAI to E-[CO_2_] was generally below 10% (Fig. 6d)^32,33^, despite increased tiller numbers. Likewise in the SPAR chamber experiments, leaf area around flowering in E-[CO_2_] (averaged for 500 and 660 µmol mol^−1^) was greater than the control by about 6%^16^. For rice, laminar length becomes shorter under E-[CO_2_]^34^, which offsets the increase in the total leaf number. It is worth noting, however, that there are certain species that show substantially greater response of LAI to E-[CO_2_], such as poplar^35^ and some invasive weed species^36^. Leaf area expansion for these species is likely driven by carbon supply. On the other hand, rice is generally conservative in leaf morphology and expansion as a function of [CO_2_], and is mostly limited by N supply and temperature. Modern cultivars of major crops such as rice may have been bred to be less plastic morphologically in response to excess amounts of resources, in order to be adapted to increased fertilization and dense planting. In previous studies comparing old and new cultivars of wheat and oats^37,38^, morphological plasticity of modern cultivars in response to increased carbon gain has been reported to be less than that of cultivars released prior to the Green Revolution.

The weak correlation to biomass changes under sub-ambient [CO_2_] conditions (Figure S9d,e) suggests the modest importance of carbon as a resource to determine biomass responses. Here we show a strong need to improve the simulation of leaf area production in response to E-[CO_2_] to reduce uncertainties in predicting yield under E-[CO_2_], which is more important than the types of photosynthetic responses to elevated [CO_2_].

It is generally reported that N availability limits biomass response to elevated [CO_2_]^39,40^. In the rice FACE experiments, the observed yield response to E-[CO_2_] was indeed limited under low N conditions^22^. Final biomass increased in E-[CO_2_] in the FACE experiments, even in the low N treatment^32^, but harvest index decreased to reduce the positive effects of E-[CO_2_] on grain yield^41^. The models failed to reproduce the limited response to E-[CO_2_] at low N. Models differ in the way they treat the effect of N on biomass production and partitioning to grains, in particular under E-[CO_2_], which resulted in a large variation in simulated yield response to E-[CO_2_] under low N conditions both in the FACE and SPAR chamber experiments (Figs 5 and S5). There is a strong need to improve the accuracy of modeling [CO_2_] and N interactions because N fertilization strategies are key issues for climate change adaptation. We need further experimental evidence on how dry matter partitioning is affected by E-[CO_2_] under N-limited conditions to reduce model uncertainty.

This study shows that biomass response, and not harvest index, is the major source of between-model variability in yield response to E-[CO_2_]. Previous experimental observations showed that response of harvest index to elevated [CO_2_] varied depending on the extreme temperature conditions around flowering and grain filling^20,42^. In the two FACE and one SPAR chamber experiments used in this study, temperatures at the critical period did not exceed the upper or lower thresholds triggering severe stresses on grain setting, which in turn could reduce harvest index. Currently, only a few models account for any potential direct effect of E-[CO_2_] on grain-setting or harvest index (Table 1). We need to test model uncertainty under extreme temperatures, combined with different temperature and [CO_2_] regimes, against different data sets that cover a range of temperature conditions.

In conclusion, predictions of rice yield and biomass in response to E-[CO_2_] varied significantly among the 16 rice models - the variation was much greater than the experimental variation observed in the FACE and SPAR chamber experiments. Variation was not random; models that tended to overestimate the responses in one environment tended to overestimate in the other environments. We hypothesized that the variation between models could be accounted for by the type of algorithm used to describe photosynthetic or RUE response to E-[CO_2_], because this is the only mechanism by which models take account of the direct effects of E-[CO_2_]. However, the results showed that the variation in simulated yield was not associated with the primary response to E-[CO_2_], but was significantly associated with the variation of modelled CO_2_-related responses in LAI. This suggests that “modelled” secondary or indirect effects of E-[CO_2_] on morphological development are the sources of uncertainty. In reality, rice morphology and LAI are conservative in response to changes in E-[CO_2_]. Accounting for this more conservative nature in the models may reduce the uncertainty in biomass and yield prediction. Nitrogen levels (particularly under limited N) make the prediction more uncertain. Improving models to account for the [CO_2_] × N interaction is therefore recommended to better evaluate management practices under climate change.

## Materials and Methods

### Experimental data

Data provided for the FACE studies were obtained from two rice FACE sites in Asia (Table S1); the Shizukuishi site (N39°38′, E140°57′) located in northern Japan and the Wuxi site (N 31°37′, E 120°28′) in central eastern China, both representing the typical rice growing regions in their respective countries. Details of experimental procedures are given in Okada *et al*.^43^ and Kim *et al*.^22^ for the Shizukuishi site and in Liu *et al*.^44^ and Yang *et al*.^33^ for the Wuxi site, also summarized in Table S1. Briefly, the FACE facilities were established in farmers’ fields, and CO_2_ concentration in octagonal plots (ring, hereafter) measuring 12 m in diameter was increased by fumigating CO_2_ through emission tubes installed on the peripheries of the rings. Pure CO_2_ was released from the windward side, with the proportional–integral–derivative algorithm controlling the pressure of CO_2_ release^43^. Target [CO_2_] at both sites was 200 μmol mol^−1^ above the ambient [CO_2_].

Within the CO_2_ treatments, three levels of N fertilizer were included; low, standard and high, with the standard being similar to farmers’ practice at each site. Because conventional (or standard) N input differed greatly between the two sites, levels of N inputs also differed substantially; total N applied at the Shizukuishi site ranged from 40 to 150 kg ha^−1^, whereas applied N at the Wuxi site ranged from 150 to 350 kg ha^−1^. Eighty-three percent (in Shizukuishi) and 60% (in Wuxi) of the total N was applied as a basal dressing before transplanting and as a side-dressing at early tillering, with the remaining N side-dressed at panicle initiation. Cultivars were Akitakomachi at the Shizukuishi site and Wuxianjing 14 at the Wuxi site, which are both *japonica* types, planted widely in the respective regions. Seedlings were transplanted at 4–5 leaf age and grown under flooded conditions until 10–15 d before harvest. The planting density was 19 and 24 hills m^−2^ at Shizukuishi and Wuxi, respectively, with three seedlings per hill at both sites.

The chamber experimental data were from 1 m × 2 m sunlit, Soil Plant Atmosphere Research (SPAR) chambers with high transmission of photosynthetically-active radiation^45^ located in Gainesville, Florida, USA (N 29°37.8′, W 82°22.2′). Radiation transmission was about 100%, especially considering that irradiance would also have a higher diffuse fraction^45^. Details of the soil and cultivation practices are given by Baker *et al*.16. Briefly, the soil was a Candler fine sand (sandy, siliceous, hyperthermic uncoated Typic Quartzsamments) placed in the soil base 60 cm deep, with 50 cm of soil with a 5-cm paddy flood depth. There were no percolation losses of water or N, as the chamber base was sealed. The crop was direct-seeded (over-seeded and thinned), grown under a constant 30 °C for the first 7–10 days until flood paddy and temperature-CO_2_ treatments were established. The row spacing was 18 cm.

Response to [CO_2_] levels was tested with the following three chamber experiments using IR30, which is an *indica* cultivar. In Experiments 1 and 2^16^, plants were grown at six levels of [CO_2_] at 160, 250, 330, 500, 660, and 900 μmol mol^−1^ at a constant day/night air temperature of 31 °C and water temperature of 27 °C. Fertilization applications were aimed to be sufficient (up to 50% more than required for maximum growth). Nitrogen in the form of urea was applied in 4 splits; immediately prior to establishing the flood depth and driven into the soil by flood application (thus volatilization losses of N should be very limited). A total of 290 kg ha^−1^ of N was applied for all treatments. In Experiment 3^24,46^, plants were grown at two levels of [CO_2_] at 330 and 660 μmol mol^−1^ at day/night air temperatures of 28 °C/21 °C and at a constant water temperature of 25 °C. At each [CO_2_] level, three levels of N were imposed; 0, 100 and 200 kg ha^−1^ in 3 splits.

### Crop models and model simulations

Sixteen rice models were used in this study, but not all models simulated each experimental setup; 14 rice models were tested against the FACE data and 15 against the SPAR data (Table S2). Detailed descriptions of the models are available in the references specified in the table and a summary or classification of the models can be found in Li *et al*.^12^ and Confalonieri *et al*.^13^. All models take into account the effects of [CO_2_] either on leaf-level [CO_2_] assimilation or canopy-level conversion of radiation to biomass, and can be classified into two groups; the first uses single-leaf photosynthesis with multiple canopy layers and the second uses canopy-based radiation conversion or canopy photosynthesis by one big-leaf (Table 1). Models in the single-leaf photosynthesis group can further be classified into two categories; one that uses an empirical light response curve modified by [CO_2_] (LRC) and the other that uses the Farquhar *et al*. model^9^ (FvCB). Many models account for other direct effects of [CO_2_] such as stomatal conductance and a few models also include direct effects of [CO_2_] on phenology and spikelet sterility.

Models also have large differences in the treatment of leaf area development, which is an important growth process that strongly affects radiation interception and biomass production. In this study, we classified the leaf area production submodules into two categories; (1) leaf area increase limited by resources such as carbon and/or nitrogen allocated to the leaves and (2) leaf area increase independent of resource availability. The first type is subdivided into C-limited and N-limited models, although some models include dependence on both C and N. The second type often uses developmental stage or a temperature function to determine leaf area increase.

Specific simulation procedures for the FACE and SPAR chamber experimental conditions are described in supplementary materials and outlined below.

#### Model simulations for the FACE experiments

Each modeling group received calibration data that covers all three N levels with both ambient and elevated [CO_2_] treatments (Shizukuishi in 1999 and Wuxi in 2002). These data sets were used to test and improve model performance under E-[CO_2_]. Each group could determine how to use the available data to perform calibration or modify the functions, and the final model equations and parameters were recorded and reported to the AgMIP-Rice team leader. Then the same final model, except for cultivar and soil parameters that were site dependent, was run for all N × [CO_2_] combinations of field experiments conducted in 1998 and 2000 at Shizukuishi, and in 2001 and 2003 at Wuxi.

#### Model simulations for the SPAR experiments

Each modeling group received all required crop management, soil and weather data, phenology dates (anthesis and maturity), seasonal biomass accumulation and yield for the “ambient” [CO_2_] (330 µmol mol^−1^) treatment for all experiments. Modellers set life cycle and productivity traits for the ambient treatment, starting with the calibrated model based on FACE experiments. The model calibrated using the ambient SPAR treatment was then used to simulate all the [CO_2_] levels and temperature × [CO_2_] treatments of the SPAR study. For each modelling group, the model used to simulate the FACE and SPAR experiments used the same functions and parameters for the [CO_2_] response.

FACE and SPAR experiments used different ambient control [CO_2_]. To compare the enhancements by E-[CO_2_] between the two types of experiments, response curves for the SPAR results were first estimated using non-linear regression for each output. Using those response curves, the output at the average ambient [CO_2_] of the two FACE experimental sites (367 µmol mol^−1^) was derived. Relative increases of the SPAR results were calculated based on those estimated reference values.

### Data availability

The datasets analysed and all modifications made to individual models during the current study are available from the corresponding authors on reasonable request.

## Electronic supplementary material

Supporting information
