# Supplementary material for: Causes of variation among rice models in yield response to CO2 examined with Free-Air CO2 Enrichment and growth chamber experiments
Source: Sci Rep. 2017 Nov 1;7:14858. doi: 10.1038/s41598-017-13582-y (PMC5666007; doi:10.1038/s41598-017-13582-y)
Supplement: Supplementary file 1 — Supporting information [file 41598_2017_13582_MOESM1_ESM.pdf]

Supporting information

Causes of variation among rice models in yield response to CO_2 examined with Free-Air CO_2 Enrichment and growth chamber experiments

Toshihiro Hasegawa[1], Tao Li[2], Xinyou Yin[3], Yan Zhu[4], Kenneth Boote[5], Jeffrey Baker[7], Simone Bregaglio[8], Samuel Buis[9], Roberto Confalonieri[10], Job Fugice[11], Tamon Fumoto[12], Donald Gaydon[13], Soora Naresh Kumar[14], Tanguy Lafarge[6], Manuel Marcaida III[2], Yuji Masutomi[15], Hiroshi Nakagawa[12], Philippe Oriol[6] , Françoise Ruget[9], Upendra Singh[11], Liang Tang[4], Fulu Tao[16,17], Hitomi Wakatsuki[12], Daniel Wallach[18], Yulong Wang[19], Lloyd Ted Wilson[20], Lianxin Yang[19], Yubin Yang[20], Hiroe Yoshida[12], Zhao Zhang[21], Jianguo Zhu[22]

[1] Tohoku Agricultural Research Center, National Agriculture and Food Research Organization, Morioka, Iwate, 020-0198, Japan.
[2] International Rice Research Institute, DAPO Box 7777, Metro Manila, Philippines.
[3] Centre for Crop Systems Analysis, Wageningen University & Research, Wageningen, PO Box 430, The Netherlands.
[4] National Engineering and Technology Center for Information Agriculture, Jiangsu, Collaborative Innovation Center for Modern Crop Production, Nanjing Agricultural University, Nanjing, Jiangsu, 210095, P.R.China.
[5] University of Florida, Gainsville, Florida, 32611-0500, USA.
[6] Cirad, UMR AGAP, F-34398 Montpellier, France.
[7] United States Department of Agriculture, Agricultural Research Service, Big Spring, Texas, 79720, USA.
[8] CREA, Research Center for Agriculture and Environment, Italy.
[9] INRA, UMR1114 EMMAH, F-84914 Avignon, France
[10] University of Milan, Cassandra lab, Milan, Italy.
[11] International Fertilizer Development Center, Muscle Shoals, Alabama, 35662, USA.
[12] Institute for Agro-Environmental Sciences, National Agriculture and Food Research Organization, Tsukuba, Ibaraki, 305-8604, Japan
[13] CSIRO Agriculture and Food, St Lucia, QLD, 4067, Australia.
[14] Indian Agricultural Research Institute, New Delhi, 110012, India
[15] Ibaraki University, College of Agriculture, Inashiki, Ibaraki, 300-0393, Japan.
[16] Chinese Academy of Sciences, Institute of Geographical Sciences and Natural Resources Research, Beijing, 100101, P.R. China.
[17] Natural Resources Institute Finland (Luke), FI-00790 Helsinki, Finland
[18] INRA, UMR AGIR, Castanet Tolosan, France
[19] Yangzhou University, Hanjiang, Yangzhou, Jiangsu, 225009, China
[20] Texas A&M AgriLife Research Center, Beaumont, Texas, 77701, USA.
[21] State Key Laboratory of Earth Surface Processes and Resource Ecology, Beijing Normal University, Beijing, 100875 P.R. China.
[22] State Key Laboratory of Soil and Sustainable Agriculture, Institute of Soil Sciences, Chinese Academy of Sciences, Nanjing 210008, China

Corresponding authors:
Toshihiro Hasegawa.
Agro-environmental Research Division
Tohoku Agricultural Research Center, NARO
4 Akahira, Shimokuriyagawa, Morioka, Japan 020-0198
Tel: +81-19-643-3462
Fax: +81-19-641-7794
E-mail: thase@affrc.go.jp

Tao Li
International Rice Research Institute
DAPO Box 7777, Metro Manila, Philippines
Tel: +63(2)-580-5600
Fax: +63(2)-580-5699
Email: t.li@irri.org

Keywords: AgMIP, Free-Air CO_2 enrichment, *Oryza sativa*, Photosynthesis, Radiation use efficiency, SPAR chamber, Uncertainties, Rice simulation model

Table S1. Summary of the experimental conditions for the Japanese and Chinese FACE experiments

	Japan (1998-2000)				China (2001-2003)			
Site description								
Latitude/Longitude	39°38’N/ 140°57’E				31°37’N/120°28’E			
Soil type and properties	Andosol				Stagnic anthrosol			
	Sand 43.1%; Silt 31.4%; Clay 25.5%				Sand 9.2%; Silt 65.7%; Clay 25.1%			
	Bulk density 0.73 g cm^{-3}				Bulk density 1.2 g cm^{-3}			
	Plow layer 12.3 cm				Plow layer 13.0 cm			
	Total C 82.5 g kg^{-1}; Total N 18 g kg^{-1}				Total C 15.0 g kg^{-1} ; Total N 1.59 g kg^{-1}			
Cultivar characteristics and growing conditions								
Cultivar		Akitakomachi					Wuxiangjing 14	
Mean growth duration		146 d				158 d		
Spikelets per panicle		81					155	
Daylight hour CO_2 concentrations (μmol mol^{-1}) at the center of the plot								
Year	*1998*	*1999*	*2000*	*Mean*	*2001*	*2002*	*2003*	*Mean*
FACE plots	599	568	559	575	578	562	574	571
Ambient plots	368	369	365	367	354	364	375	364
N application rates (g N m-2)	*1998*	*1999*	*2000*		*2001*	*2002*	*2003*	
Low	4	4	4		15	15	15	
Standard	8	9	9		25	25	25	
High	12	15	15		-	35	35	
Cultural practices								
Sowing dates	1 May	23 April	23 April			18 May		
Transplanting dates	21 May	20 May	22 May			13 June		
Planting density		19 hills m^{-2}				24 hills m^{-2}		
Mean daily temperatures (°C)								
	1998	*1999*	*2000*	*Mean*	*2001*	*2002*	*2003*	*Mean*
Transplanting to tillering	15.4	17.2	17.7	16.8	26.7	24.9	25.8	25.8
Tillering to panicle initiation	19.9	20.6	21.3	20.6	29.1	28.6	29.4	29.0
Panicle initiation to heading	21.4	25.9	24.7	24.0	26.6	26.6	28.5	27.2
Heading to mid-ripening	21.3	22.5	23.6	22.5	24.1	25.7	26.0	25.3
Mid-ripening to maturity	20.3	19.8	20.9	20.3	20.9	20.3	18.8	20.0
Season mean	19.7	21.0	21.4	20.7	25.2	24.7	25.4	25.1

Mean daily solar radiation (MJ m^{-2} d^{-1})								
Transplanting to tillering	14.8	20.3	20.3	17.5	16.7	14.9	12.4	14.7
Tillering to panicle initiation	13.9	15.1	16.8	14.4	22.1	19.4	17.1	19.5
Panicle initiation to heading	14.7	19.4	17.3	16.5	17.1	14.1	15.0	15.4
Heading to mid-ripening	12.3	10.5	18.2	13.2	17.2	14.7	12.5	14.8
Mid-ripening to maturity	9.7	11.4	9.4	9.1	15.7	13.0	10.9	13.2
Season mean	13.2	16.2	16.8	14.6	17.6	14.9	13.3	15.2

64

Methods for uncertainty quantification

Simulations of growth and yield include two sources of uncertainty. One is the uncertainty caused by the difference between measured and simulated yield (Ubias) and the other is due to the variation among crop models (Umodel). In this study, we quantified both uncertainties by the following two methods:

1. To determine overall Ubias, a pairwise comparison between observed and simulated was made for datasets excluding those used for the model calibration. This was followed by further analysis on the deviations between simulations and observations to quantify the source of Ubias.
2. To determine sources of Umodel, the sum of squares (SS) from different sources was calculated for all simulation results.

FACE study

Simulations vs. observations

In step 1, we compared the measured and simulated yield and biomass under ambient [CO_2]. The bias for grain yield in Shizukuishi was significantly different from 0 (P<0.05), but only about 3.6 % of the measured yield. In Wuxi, simulated and measured yield did not differ significantly, but biomass was underestimated by 1.2 t/ha in Wuxi (P < 0.001), but about 6.7 % of the measured mean of 17.3 t/ha. The bias was consistent over the N treatments (Table S3, Figure S2). The mean bias in biomass of 0.4 t/ha in Shizukuishi was not significant.

The mean enhancement of grain yield in response to elevated [CO_2] was slightly but significantly overestimated in Shizukuishi, by 3 percentage point ($P<0.05$, Table S3, Figure S3). The bias was greater under low or high N conditions. In Wuxi, there was virtually no bias observed when averaged, but was slightly overestimated in medium N but underestimated in high N (Table S3, Figure S3). Overall, however, the mean biases were mostly not greater than the range of experimental errors (Table S3), suggesting that the mean of models was close to the measurements. We further analysed the sources of variation in the biases by means of analysis of variance, using differences between measured and simulated as a dependent variable and N and models as independent variables (Table S4). In general, more than half of the variation was due to models. These results suggest that Ubias is not large for the model ensemble means, but that for individual models is not negligible.

Variation among models (Umodel)

The simulated yield and biomass were further analysed for their sources of variation in a similar manner to the previous variance components (Table S5). Here we used all datasets including those used for calibration because the analyses targeted variation only among the simulated data. Variation in the experimental data is also shown in the table for reference, however. For simulated yield and biomass, models are a large source of variation, but variation due to year and nitrogen levels were also large sources. The modelled variation follows that in observed yields and biomass. Model by N interactions were relatively small, suggesting that the models tend to behave similarly to different levels of N. The results were similar in both sites.

For yield and biomass enhancements to elevated [CO_2], 60% or more of the variation was due to the models. The main effect of year or N did not account for the variation (7 % or less).

SPAR chamber study

A pairwise comparison between the simulated and observed was first made on the yield and biomass under ambient (330 μmol mol^{-1}) conditions, using Experiment 1, 2 and 3 under sufficient N conditions (Table S6). For the SPAR study, models were not calibrated except for phenology so all the data were used for this comparison. On average, grain yield was slightly but consistently overestimated by the models but no significant difference was observed for biomass. Models account for 65 % and 55 % of the total variation in the model bias for grain yield and biomass respectively.

Grain yield enhancement was significantly underestimated by the models ($P < 0.05$) by 4.5 percentage points and biomass was overestimated by 3.8%, but these differences were less than the standard error for the experiments (Table S6). The major source of the variation in the model simulations was models, accounting for more than 60 % of the total variations (Table S7). The model by CO_2 interaction was relatively small ranging from 4 to 6 %, suggesting that relative model performance was similar under two [CO_2] conditions.

These analyse highlight the importance of reducing uncertainties among the models.

Table S2 A paired T-test comparing measurements with simulated yield and aboveground biomass under ambient [CO_2] and enhancement by elevated [CO_2] in the FACE experiments with three N fertilizer levels. The datasets used for model calibration are not included.

Site		Mean difference (measured-simulated)	df	t	P value	SE for the measurements[1)]	Difference (measured-simulated) in each N treatment					
							low N		medium N		high N	
Shizukuishi	Grain yield (t/ha) (ambient)	0.3	82	-2.39	0.019	0.63	0.4		0.0		0.4	
	Biomass (ambient) (t/ha)	0.4	71	-1.43	0.156	0.89	1.0	a	0.5	ab	0.2	bc
	Grain yield enhancement (%)	-3.0	82	2.50	0.015	9.0	-5.3	a	1.0	b	-4.5	a
	Biomass enhancement (%)	-4.1	71	3.04	0.003	10.9	-3.5		-5.9		-4.2	
Wuxi	Grain yield (ambient) (t/ha)	0.2	57	-1.11	0.271	0.37	0.8	a	0.4	ab	-0.9	bc
	Biomass (ambient) (t/ha)	1.2	57	-4.30	0.000	0.68	1.1		1.5		0.5	
	Grain yield enhancement (%)	-0.9	57	0.83	0.408	7.5	-0.7	ab	-3.4	a	2.6	c
	Biomass enhancement (%)	3.7	57	-2.97	0.004	8.8	9.2	a	-4.3	c	6.0	b

1) Standard error for the measurements was obtained from the residual mean square of the analysis of variance using year and N as main factors (see Table S3).

2) The same alphabetic letters followed by the values for each N treatment are not significantly different between the treatments.

Table S3. Sources of variation in the difference between measured and simulated values in the FACE experiments.

Site	Source	Grain yield (ambient) (t/ha)^2				Biomass (ambient) (t/ha)^2				Yield enhancement (%)^2				Biomass enhancement (%)^2			
		df	SS	MS	%2)	df	SS	MS	%	df	SS	MS	%	df	SS	MS	%
Shizukuishi	N level	2	2.4	1.2	2.6	2	8.3	4.1	2.6	2	506	253.0	5.0	2	93	46	1.0
	Model	13	63.1	4.9	66.4	13	262.1	20.2	81.7	13	6376	490.5	63.3	13	6410	493	67.4
	Model × N level	20	4.1	0.2	4.3	20	19.1	1.0	6.0	20	1201	60.0	11.9	20	628	31	6.6
	Error	47	25.4	0.5	26.7	36	31.3	0.9	9.8	47	1998	42.5	19.8	36	2373	66	25.0
	Corrected Total	82	95.0			71	320.8			82	10081			71	9504		
Wuxi	N level	2	26.3	13.1	20.2	2	7.2	3.6	3.3	2	317	158.4	7.5	2	1899	949	45.9
	Model	13	84.5	6.5	65.1	13	128.3	9.9	58.7	13	2128	163.7	50.6	13	1488	114	36.0
	Model × N level	20	10.1	0.5	7.8	17	12.8	0.8	5.8	20	822	41.1	19.6	17	622	37	15.0
	Error	22	9.0	0.4	6.9	19	70.4	3.7	32.2	22	937	42.6	22.3	19	127	7	3.1
	Corrected Total	57	129.9			51	218.7			57	4204			51	4136		

1) Sum of squares (SS) for the differences between measured and simulated values were calculated by the general liner model procedures (Type I sum of squares).

2) % of SS of the total (corrected by the overall mean).

Table S4 Sum of squares (SS) and mean squares (MS) of the simulated and measured yield and biomass under ambient [CO_2] and of enhancement due to elevated [CO_2] at two FACE sites.

			Yield (ambient) (t/ha)^2				Biomass (ambient) (t/ha)^2				Yield Enhancement (%)^2				Biomass Enhancement (%)^2			
Site		Source	df	SS	MS	% 1)	df	SS	MS	%	df	SS	MS	%	df	SS	MS	%
Shizukuishi	Simulated	Year	2	44.1	22.03	21.4	2	108	53.8	15.9	2	904	452	6.7	2	903	451.3	7.2
		Nitrogen (N)	2	63.7	31.84	31.0	2	198	98.8	29.2	2	821	410	6.1	2	586	293.2	4.7
		Year × N	4	3.23	0.81	1.6	4	7.41	1.9	1.1	4	177	44	1.3	4	49.7	12.4	0.4
		Model	13	64.1	4.93	31.2	13	291	22.4	42.9	13	8006	616	59.6	13	8716	670.5	69.4
		Model × Year	26	23.2	0.89	11.3	26	47.1	1.8	7.0	26	1362	52	10.1	26	1198	46.1	9.5
		Model × N	20	3.67	0.18	1.8	20	17.1	0.9	2.5	20	1126	56	8.4	20	650	32.5	5.2
		Model × Year × N	40	3.55	0.09	1.7	40	9.23	0.2	1.4	40	1034	26	7.7	40	448	11.2	3.6
		Total	107	205	1.92		107	677	6.3		107	13429	126		107	12551	117.3	
	Measured	Year	2	28.1	14.03	50.8	2	22.8	11.4	31.7	2	286	143	11.3	2	606	303	15.4
		Block[2)]	3	1.6	0.52	2.8	3	3.3	1.1	4.6	3	43	14	1.7	3	29	10	0.7
		Year × Block	6	1.5	0.26	2.8	6	5.9	1.0	8.3	6	258	43	10.2	6	947	158	24.1
		N	2	16.9	8.45	30.6	2	24.8	12.4	34.6	2	414	207	16.4	2	168	84	4.3
		Year × N	4	0.1	0.01	0.1	3	3.1	1.0	4.3	4	67	17	2.7	3	409	136	10.4
		Sub plot error[3)]	18	7.1	0.39	12.8	15	11.9	0.8	16.6	18	1454	81	57.7	15	1771	118	45.1
		Total	35	55	1.58		31	71.9	27.7		35	2520.6	504.4		31	3928.4	808.5	
Wuxi	Simulated	Year	2	50.6	25.3	22.4	2	186	92.8	34.4	2	256	128.1	4.2	2	32.2	16.1	0.62
		N	2	17.4	8.7	7.7	2	57.7	28.8	10.7	2	53.5	26.8	0.9	2	250	124.8	4.8
		Year × N	4	0.87	0.2	0.4	4	3.79	0.9	0.7	4	21.3	5.3	0.4	4	7.36	1.8	0.14
		Model	13	111	8.5	48.9	13	180	13.8	33.3	13	3775	290.4	62.1	13	3669	282.2	70.4
		Model × Year	26	32.2	1.2	14.3	26	77.2	3.0	14.3	26	862	33.1	14.2	26	412	15.9	7.9
		Model × N	20	12.3	0.6	5.5	20	31.9	1.6	5.9	20	649	32.4	10.7	20	710	35.5	13.6
		Model × Year × N	35	1.95	0.1	0.9	35	3.92	0.1	0.7	35	459	13.1	7.6	35	132	3.8	2.5
		Total	102	226	2.22		102	540	5.3		102	6076	59.6		102	5212	51.1	
	Measured	Year	2	12.23	6.11	66.3	2	5.71	2.86	20.8	2	9.7	4.8	0.9	2	64.1	32.1	2.9
		Block[2)]	2	0.06	0.03	0.3	2	0.18	0.09	0.7	2	56.9	28.5	5.0	2	250.4	125.2	11.5
		Year × Block	4	1.28	0.32	6.9	4	5.20	1.30	18.9	4	174.5	43.6	15.4	4	394.3	98.6	18.1
		N	2	3.04	1.52	16.5	2	9.13	4.56	33.2	2	140.2	70.1	12.3	2	434.7	217.3	20.0
		Year × N	3	0.44	0.15	2.4	3	2.70	0.90	9.8	3	198.7	66.2	17.5	3	255.6	85.2	11.7
		Sub plot error[3)]	10	1.38	0.14	7.5	10	4.59	0.46	16.7	10	555.9	55.6	48.9	10	778.7	77.9	35.8
		Total	23	18	0.80		23	28	1.20		23	1136	49.39		23	2178	94.69	

1) % of SS of the total (corrected by the overall mean).

2) Randomized complete block design for the N treatments each year at each site.

3) Residual variance of the experiment

Table S5. A paired comparison between measured and simulated yield and aboveground under 330 µmol mol^{-1} and between measured and simulated enhancements due to elevated CO_2 (660 & 500 µmol mol^{-1}) in the SPAR experiment

Variables compared	Mean difference (measured-simulated)	df	t	P value	Standard error for the measurements
Grain yield (t/ha) (330µmol mol^{-1})	-1.28	41	-3.41	0.001	1.29
Biomass (t/ha) (330µmol mol^{-1})	-0.21	41	-0.33	0.742	1.79
Grain yield enhancement (%)	4.47	57	2.22	0.031	5.24
Biomass enhancement (%)	-3.83	57	-2.06	0.044	6.05

Table S6. Sum of squares (SS) and mean square (MS) of yield and biomass under ambient [CO_2] (330µmol mol-1) and of enhancement due to elevated CO_2 (660 & 500 µmol mol-1) in SPAR experiment

	Grain yield (330 µmol mol^{-1}) (t/ha)^{2}				Biomass (330 µmol mol^{-1}) (t/ha)^{2}				Yield Enhancement (%)^{2}				Biomass Enhancement (%)^{2}			
Source	df	SS	MS	%	df	SS	MS	%	df	SS	MS	%	df	SS	MS	%
CO_2									1	1051	1051	11.4	1	1402	1402	14.0
Experiment	2	26.5	13.3	11.9	2	125	62.4	19.5	2	586	293	6.35	2	601	300	6.01
CO2×Experiment									1	0.554	0.554	0.006	1	3.33	3.33	0.03
Model	14	158	11.3	71.1	14	366	26.1	57.1	14	5915	422	64.1	14	6756	483	67.6
Model * CO_2									14	549	39.2	5.95	14	426	30.5	4.27
Error	25	37.8	1.51	17.0	25	151	6.02	23.5	39	1129	28.9	12.2	39	803	20.6	8.04
Corrected Total	41	222			42	12010			71	9231			71	9992		

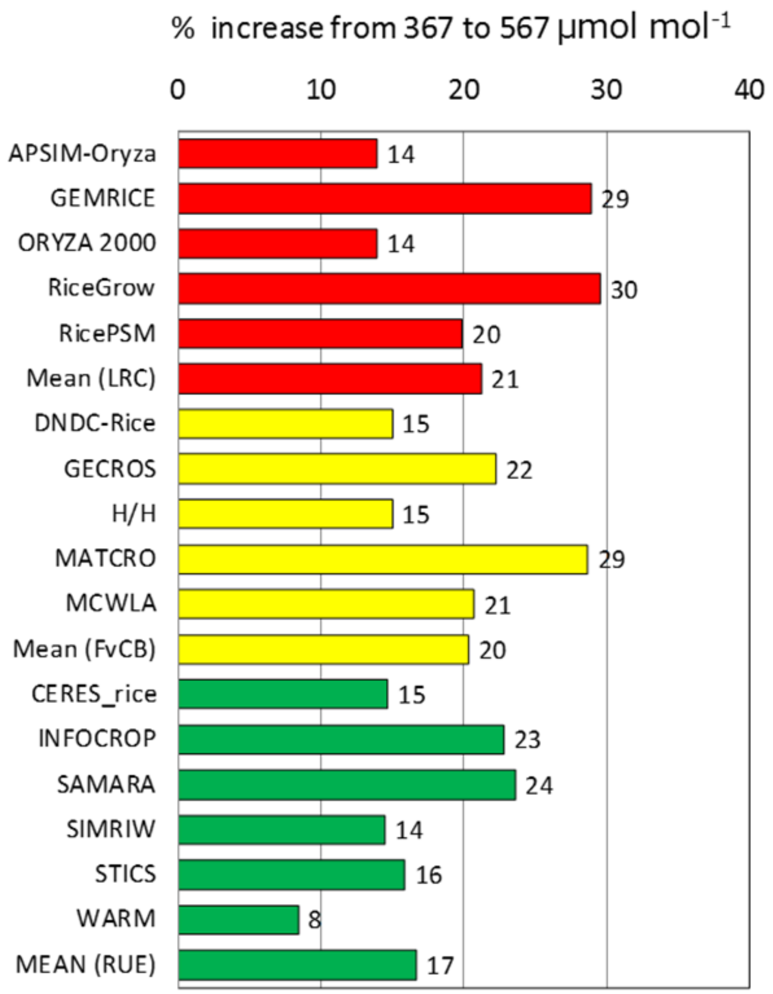

Figure S1. Responses of leaf photosynthesis or radiation use efficiency used in 16 rice models. Values expressed as % increase with a [CO_2] elevation by 200 µmol mol^{-1} from 367 µmol mol^{-1} to Red, LRC (light response curve-type photosynthesis model); Yellow, FvCB (Farquhar, von Caemmerer & Berry photosynthesis model); Green, RUE (Radiation use efficiency).
Analysis of variance testing the difference among model types showed no significant differences.

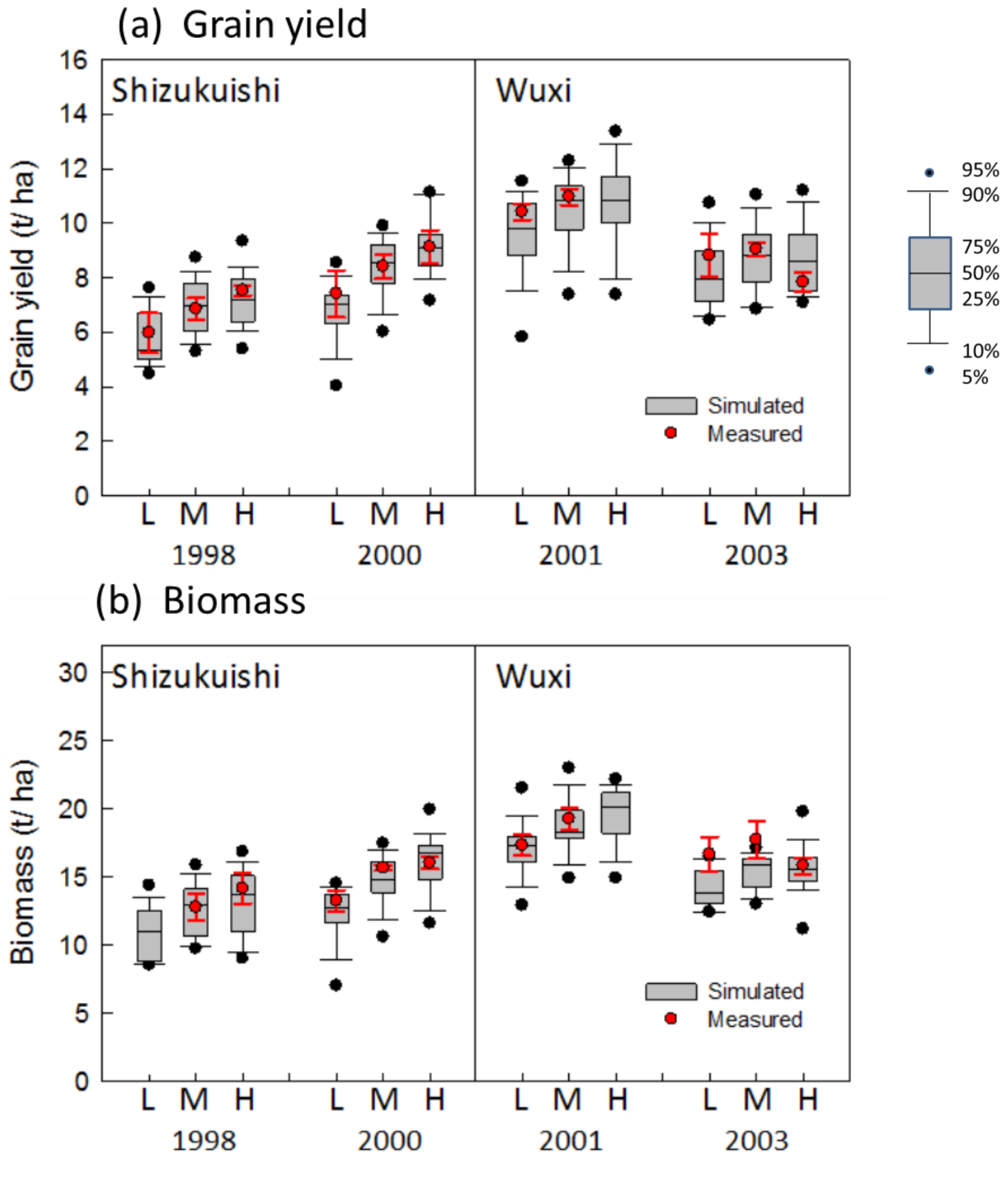

Figure S2. Simulated and observed yield (a) and biomass (b) under ambient [CO_2]
Box-whiskers represent the variation among simulated values by 14 rice models and red dots and bars represent the measured mean ±80% confidence intervals. The 80 % confidence intervals are to compare the whiskers of the simulated values ranging from 10 to 90 percentiles. Datasets not used for the calibration are shown. Measurements for biomass in LN at Shizukuishi in 1998 and for yield and biomass in HN in 2001were not available.

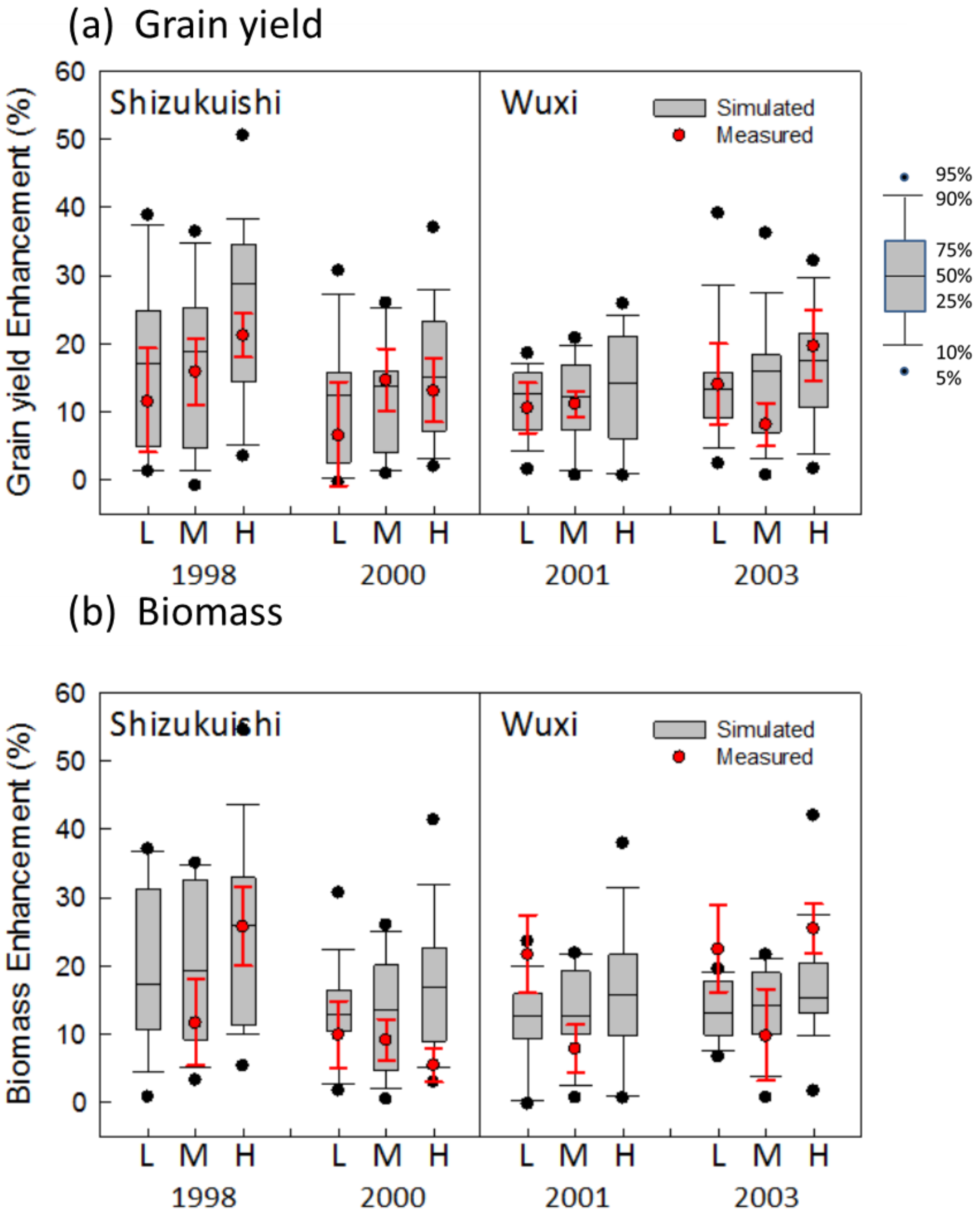

Figure S3. Simulated and observed yield (a) and biomass (b) enhancement by elevated [CO_2]
Box-whiskers represent the range of simulated values by 14 rice models and red dots and bars represent the measured mean ±80% confidence intervals. The 80 % confidence intervals are to compare the whiskers of the simulated values ranging from 10 to 90 percentiles.
Datasets not used for the calibration are shown.
Measurements for biomass in LN at Shizukuishi in 1998 and for yield and biomass in HN in 2001were not available.

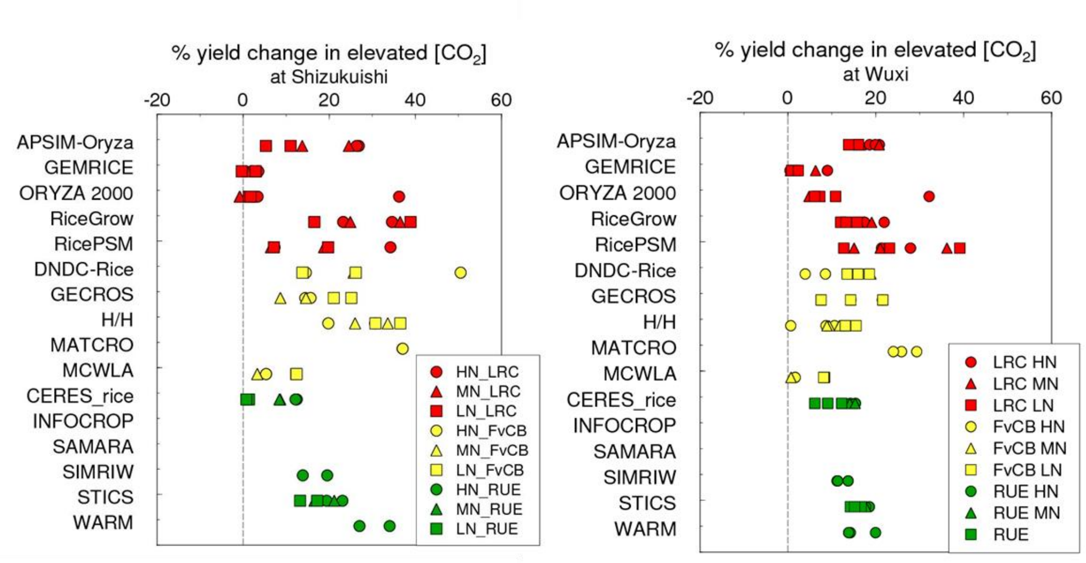

Figure S4. Simulated yield enhancements due to elevated [CO_2] for individual models at Shizukuishi (a) and Wuxi (b).

Variation within each model derives from differences in simulated yields under different N or years. Values expressed as % increase with a [CO_2] elevation by 200 µmol mol^{-1} from 367 µmol mol^{-1} to 567 µmol mol^{-1}. LRC, light response curve-type photosynthesis model; FvCB, Farquhar, von Caemmerer & Berry photosynthesis model; RUE, radiation use efficiency.

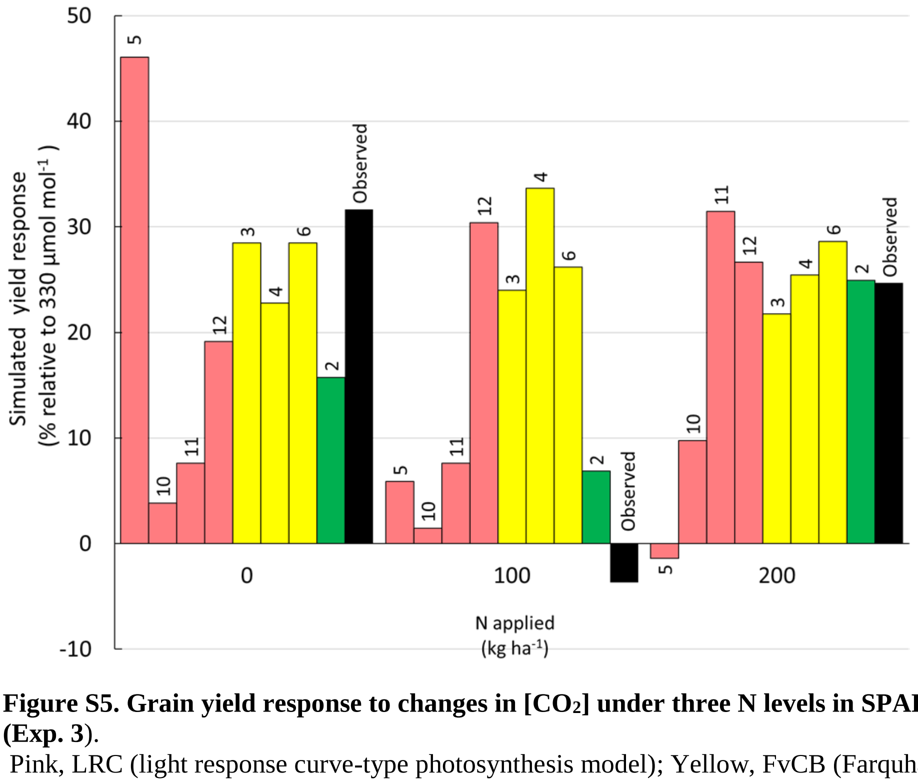

Figure S5. Grain yield response to changes in [CO_2] under three N levels in SPAR experiment (Exp. 3).

Pink, LRC (light response curve-type photosynthesis model); Yellow, FvCB (Farquhar, von Caemmerer & Berry photosynthesis model); Green, RUE (Radiation use efficiency).

Numbers above or below the bars correspond to the individual models listed in Table S1.

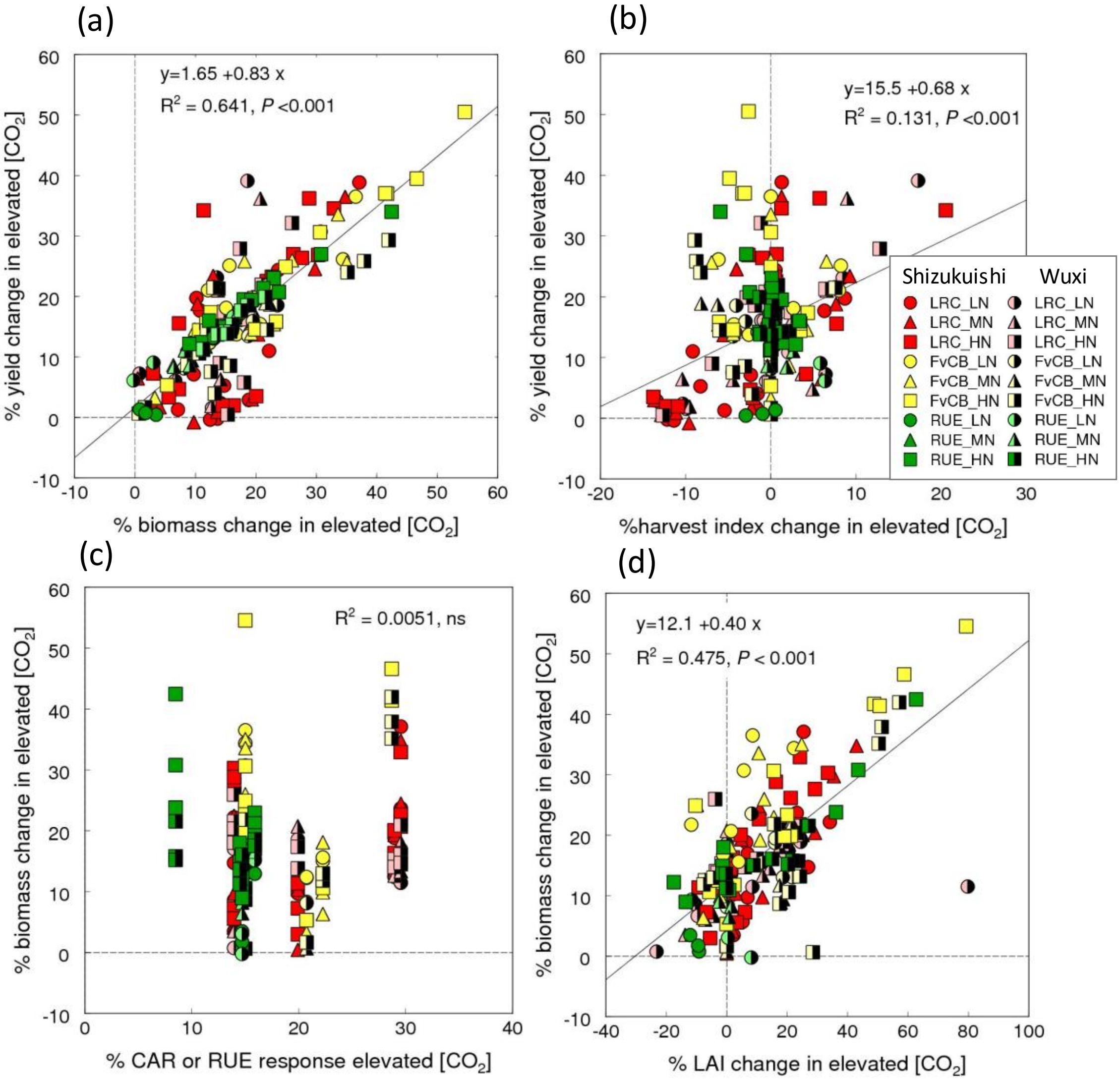

Figure S6. Factors affecting the simulated enhancements of grain yield due to E-[CO_2] in high N treatment in the FACE experiments at two sites. (a) grain yield enhancement versus biomass enhancement, (b) grain yield enhancement versus harvest index enhancement, (c) biomass enhancement versus primary [CO_2] enhancement (leaf CO_2 assimilation rate, CAR, or radiation use efficiency, RUE) , and (d) biomass enhancement versus maximum LAI enhancement. The data from the all N treatments at two sites are included. Symbols with a single color are for Shizukuishi and those that are half-filled in black are for Wuxi. LRC, light response curve-type photosynthesis model; FvCB, Farquhar, von Caemmerer & Berry photosynthesis model; RUE, radiation use efficiency.

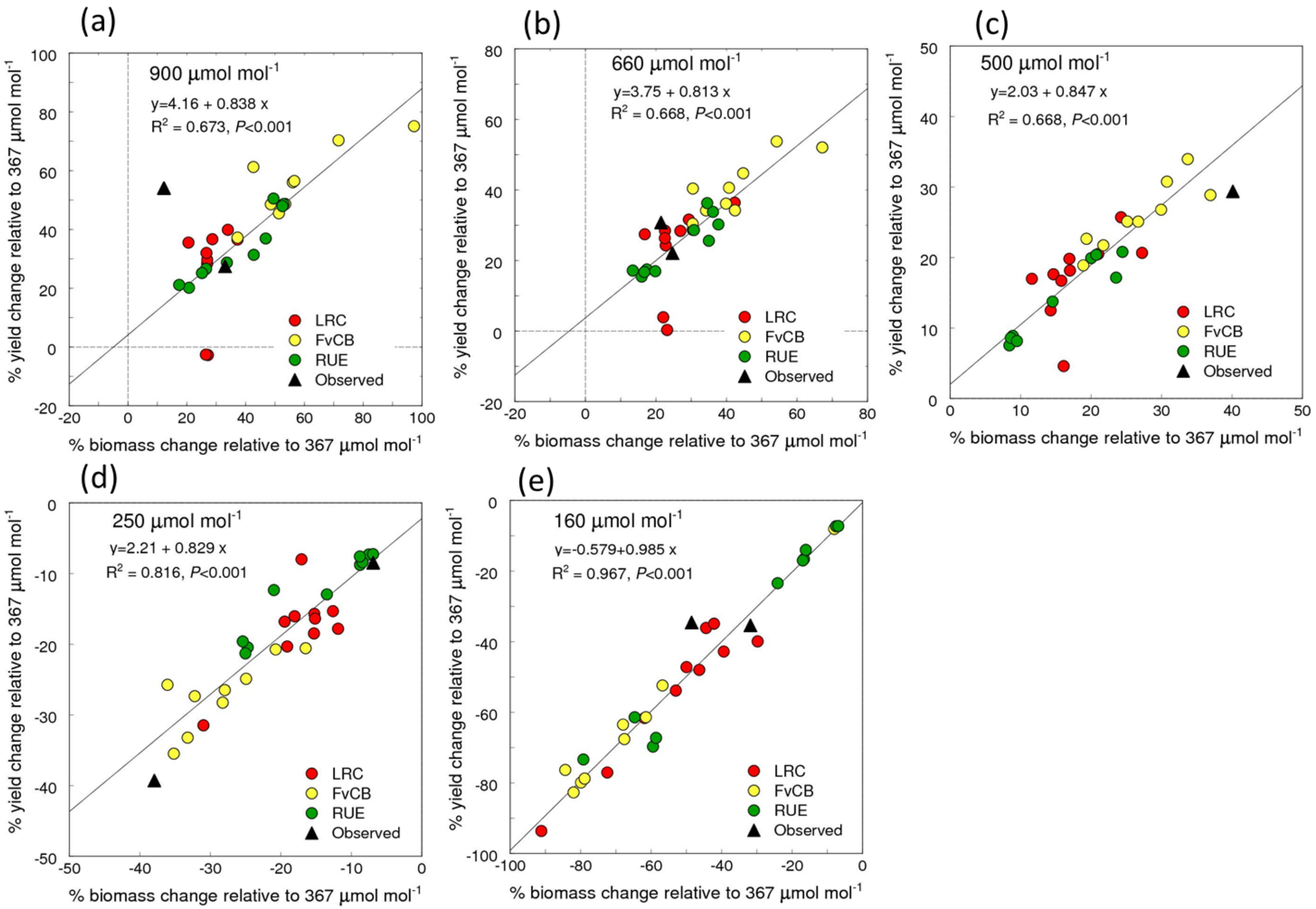

Figure S7. Relationship between grain yield and biomass responses to various levels of [CO_2] ((a) 900 µmol mol^{-1}, (b) 660 µmol mol^{-1}, (c) 500 µmol mol^{-1}, (d) 660 µmol mol^{-1}, (e)160 µmol mol^{-1}) in the SPAR chamber experiments (Ex.1 & 2).
% changes are relative to the value at 367 µmol mol^{-1}. LRC, light response curve-type photosynthesis model; FvCB, Farquhar, von Caemmerer & Berry photosynthesis model; RUE, radiation use efficiency.

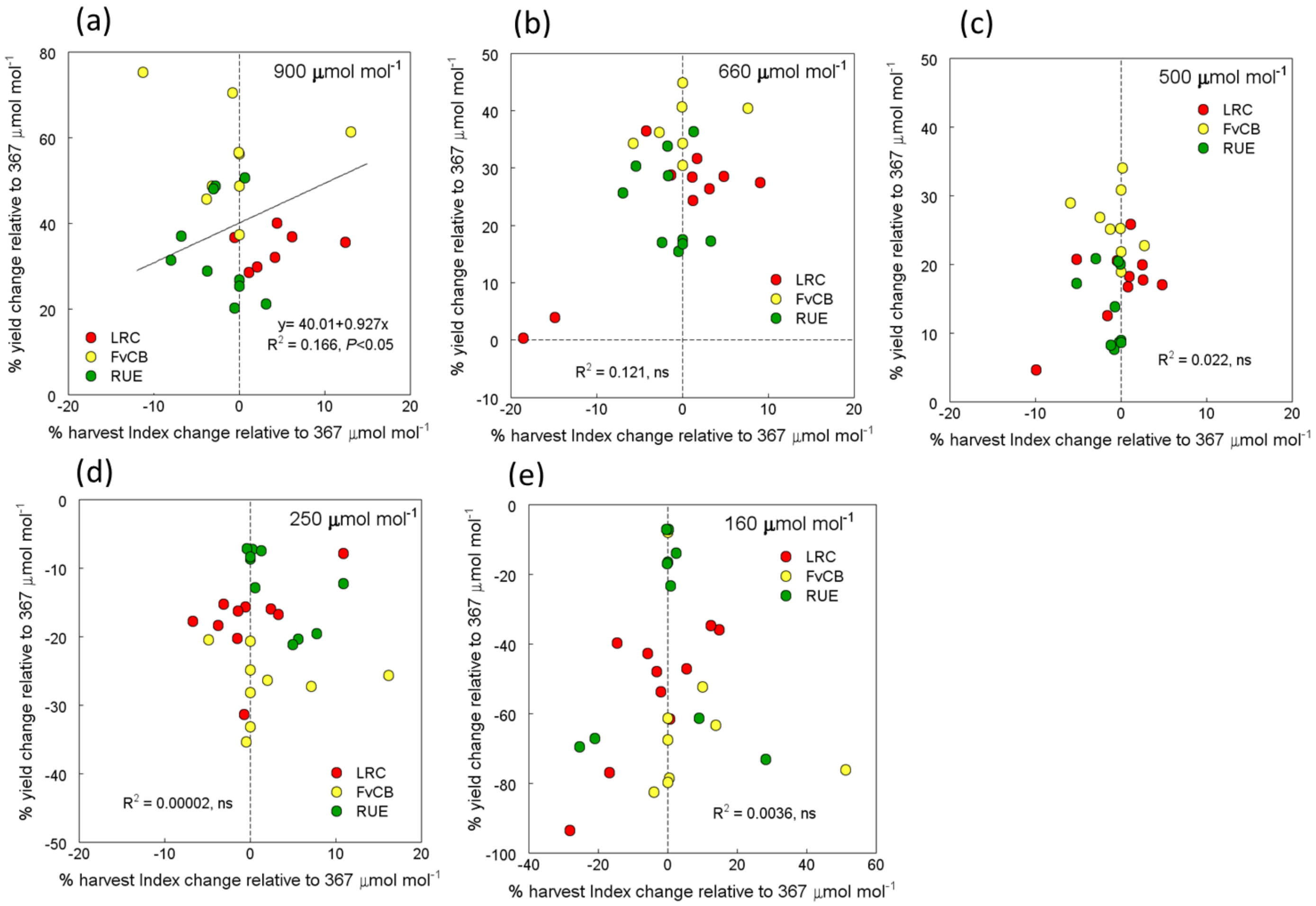

Figure S8. Relationship between grain yield and harvest index responses to various levels of [CO_2] ((a) 900 µmol mol^{-1}, (b) 660 µmol mol^{-1}, (c) 500 µmol mol^{-1}, (d) 660 µmol mol^{-1}, (e)160 µmol mol^{-1})in the SPAR chamber experiments (Ex.1 & 2).

% changes are relative to the value at 367 µmol mol^{-1}. LRC, light response curve-type photosynthesis model; FvCB, Farquhar, von Caemmerer & Berry photosynthesis model; RUE, radiation use efficiency.

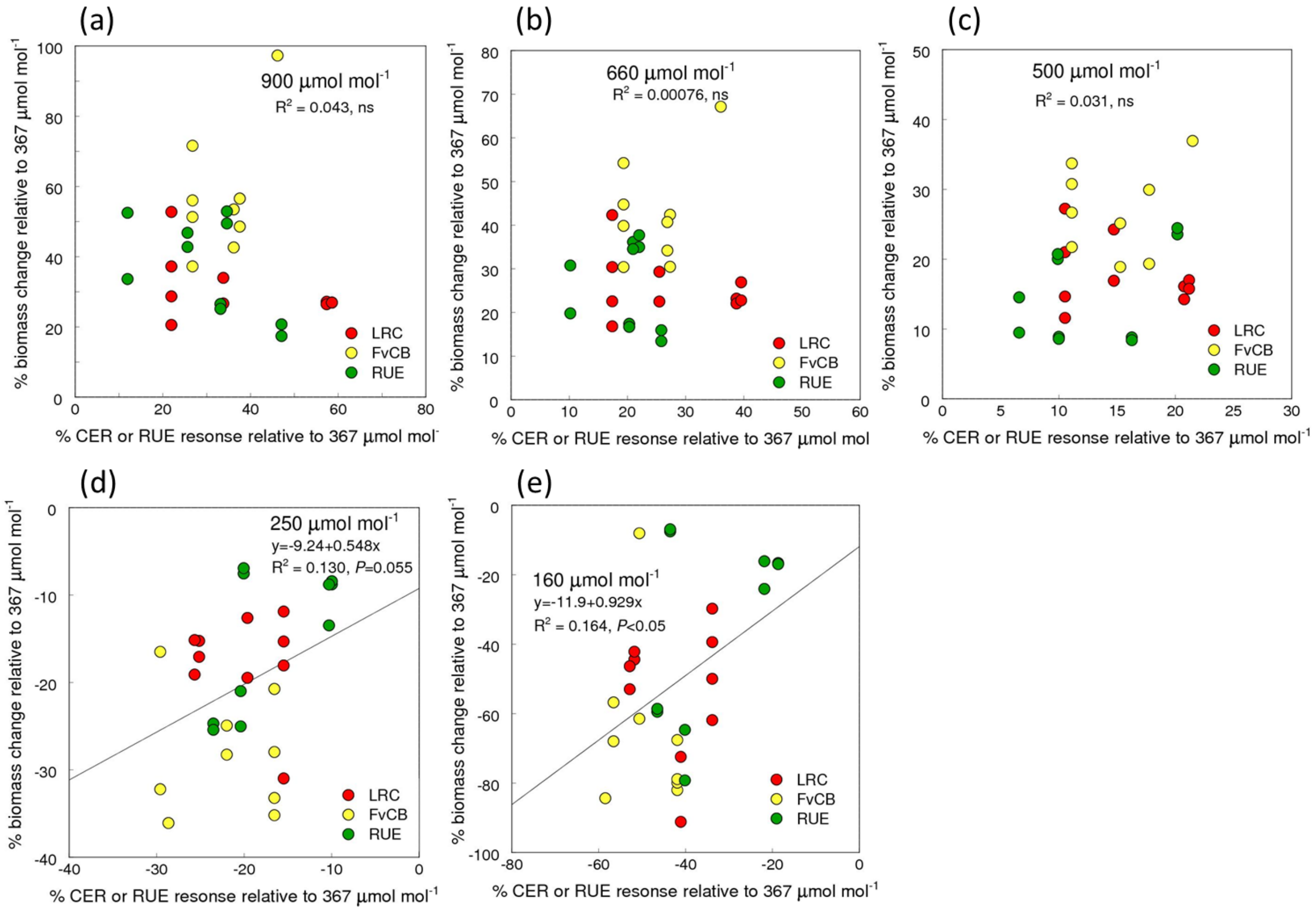

Figure S9. Relationship between primary [CO_2] of each model and biomass response to various levels of [CO_2] ((a) 900 µmol mol^{-1}, (b) 660 µmol mol^{-1}, (c) 500 µmol mol^{-1}, (d) 660 µmol mol^{-1}, (e)160 µmol mol^{-1}) in the SPAR chamber experiments (Ex 1 & 2).

% changes are relative to the value at 367 µmol mol^{-1}. LRC, light response curve-type photosynthesis model; FvCB, Farquhar, von Caemmerer & Berry photosynthesis model; RUE, radiation use efficiency.

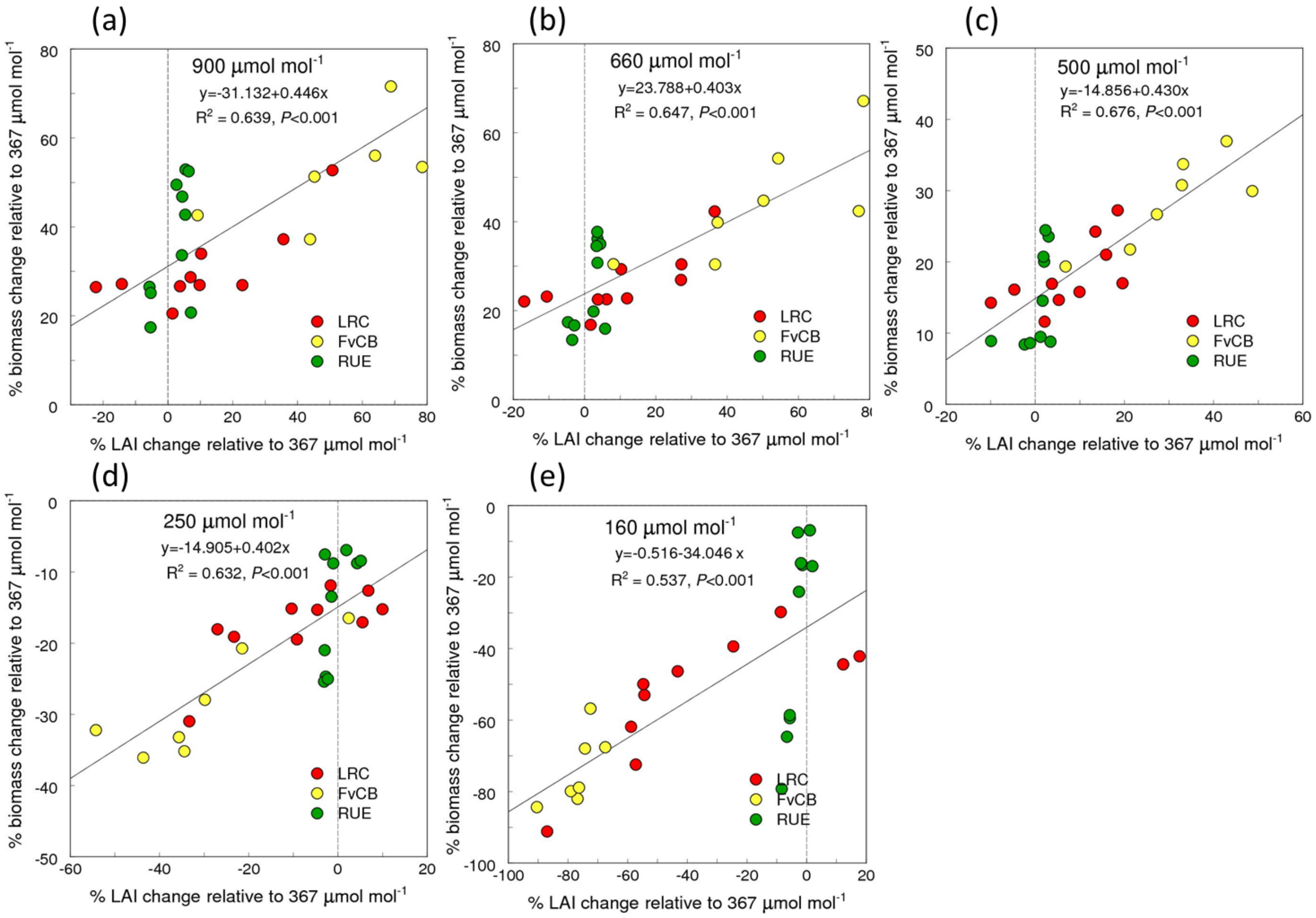

Figure S10. Relationship between maximum LAI response and biomass response to various levels of [CO_2] ((a) 900 µmol mol^{-1}, (b) 660 µmol mol^{-1}, (c) 500 µmol mol^{-1}, (d) 660 µmol mol^{-1}, (e)160 µmol mol^{-1}) in the SPAR chamber experiments (Ex 1 & 2).

% changes are relative to the value at 367 µmol mol^{-1}. LRC, light response curve-type photosynthesis model; FvCB, Farquhar, von Caemmerer & Berry photosynthesis model; RUE, radiation use efficiency.

144
